# Comparative physiological, transcriptomic, and WGCNA analyses reveal the key genes and regulatory pathways associated with drought tolerance in Tartary buckwheat

**DOI:** 10.3389/fpls.2022.985088

**Published:** 2022-10-03

**Authors:** Heng-Ling Meng, Pei-Yuan Sun, Jia-Rui Wang, Xiao-Qian Sun, Chuan-Zhi Zheng, Ting Fan, Qing-Fu Chen, Hong-You Li

**Affiliations:** ^1^ Research Center of Buckwheat Industry Technology, Guizhou Normal University, Guiyang, China; ^2^ Key Laboratory of High-Quality Crops Cultivation and Safety Control of Yunnan Province, Honghe University, Honghe, China; ^3^ College of Life Science, Guizhou Normal University, Guiyang, China; ^4^ Key Laboratory of Plant Resources Conservation and Germplasm Innovation in Mountainous Region of Ministry of Education, Guizhou University, Guiyang, China

**Keywords:** Tartary buckwheat, drought stress, transcriptome, transcription factor, transcriptional regulatory network, WGCNA

## Abstract

Drought stress is one of the major abiotic stress factors that affect plant growth and crop productivity. Tartary buckwheat is a nutritionally balanced and flavonoid-rich pseudocereal crop and also has strong adaptability to different adverse environments including drought. However, little is known about its drought tolerance mechanism. In this study, we performed comparative physiological and transcriptomic analyses of two contrasting drought-resistant Tartary buckwheat genotypes under nature drought treatment in the reproductive stage. Under drought stress, the drought-tolerant genotype XZSN had significantly higher contents of relative water, proline, and soluble sugar, as well as lower relative electrolyte leakage in the leaves than the drought-susceptible LK3. A total of 5,058 (2,165 upregulated and 2,893 downregulated) and 5,182 (2,358 upregulated and 2,824 downregulated) potential drought-responsive genes were identified in XZSN and LK3 by transcriptome sequencing analysis, respectively. Among the potential drought-responsive genes of XZSN, 1,206 and 1,274 genes were identified to be potential positive and negative contributors for XZSN having higher drought resistance ability than LK3. Furthermore, 851 out of 1,206 positive drought-resistant genes were further identified to be the core drought-resistant genes of XZSN based on WGCNA analysis, and most of them were induced earlier and quicker by drought stress than those in LK3. Functional annotation of the 851 core drought-resistant genes found that a large number of stress-responsive genes were involved in TFs, abscisic acid (ABA) biosynthesis, signal transduction and response, non-ABA signal molecule biosynthesis, water holding, oxygen species scavenging, osmotic adjustment, cell damage prevention, and so on. Transcriptional regulatory network analyses identified the potential regulators of these drought-resistant functional genes and found that the HD-ZIP and MYB TFs might be the key downstream TFs of drought resistance in Tartary buckwheat. Taken together, these results indicated that the XZSN genotype was more drought-tolerant than the LK3 genotype as evidenced by triggering the rapid and dramatic transcriptional reprogramming of drought-resistant genes to reduce water loss, prevent cell damage, and so on. This research expands our current understanding of the drought tolerance mechanisms of Tartary buckwheat and provides important information for its further drought resistance research and variety breeding.

## Introduction

Drought/water deficit is one of the most significant environmental stresses, and it restricts plant growth, development, and reproduction as well as threatens worldwide agricultural production and food safety (Thirumalaikumar et al., 2018; Waititu et al., 2021; Wang et al., 2021; Ksouri et al., 2016; Tang et al., 2022; Zhao et al., 2022). It has been shown that drought stress in the reproductive stage will cause an average crop yield reduction of more than 50% (Hu and Xiong, 2014). In addition, 64% of the worldwide land area is affected by drought stress, and the area still continues to increase due to the current ongoing climate changes (Lesk et al., 2016; Omprakash et al., 2017; Waititu et al., 2021). Consequently, dissecting the drought resistance mechanisms in plants or crops and developing drought-resistant crop varieties are the most promising solutions to maintain crop yields under drought conditions and alleviate future threats to food security (Shaar-Moshe et al., 2015; Waititu et al., 2021).

In order to cope with and adapt to drought stress, plants have evolved various drought resistance mechanisms at multiple levels such as molecular, physiological, cellular, and morphological levels (Shaar-Moshe et al., 2015; Zhu, 2016; Wang et al., 2018a). These mechanisms include promoting the formation of deeper roots to increase water uptake, closing the stomata or thickening the leaf cuticle to prevent water loss, and shortening the life cycle through accelerating flowering (Zhu, 2016; Wang et al., 2018a). At the molecular level, plants have developed abscisic acid (ABA)-dependent and ABA-independent pathways to regulate drought resistance (Shinozaki and Yamaguchi-Shinozaki, 2007; Roychoudhury et al., 2013). Among the two regulatory pathways, the ABA-dependent pathway is the major and conserved molecule signal pathway for plant drought resistance (Saradadevi et al., 2017). The ABA content increases rapidly under drought stress by quickly inducing the expression of ABA synthesis genes. As a signal molecule, the accumulated ABA is recognized by the ABA receptor PYR/PYL/RCAR and subsequently initiates the corresponding signal transduction pathways mediated by phosphatases and protein kinases, which further activate or suppress the downstream target transcription factors (TFs) such as AREB/ABF, AP2/ERF, MYB, NAC, HD-ZF, HD-ZIP, bHLH, C2H2-ZF, B3, WRKY, and NF-Y TFs (Fujita et al., 2011; You et al., 2019; Waititu et al., 2021). These TFs further activate the expression of a large number of downstream stress-response genes, leading to a series of physiological, metabolic, cellular, and morphological responses, so as to enhance the drought resistance of plants. These responses include scavenging reactive oxygen species (ROS) through enzymatic and non-enzymatic components; increasing water uptake through the generation of deeper roots; reducing the water loss of leaves through the regulation of stomatal closure or leaf cuticular wax biosynthesis; increasing the accumulation of osmoprotectants such as amino acids, glycine betaine, polyamine, and sugars to perform osmotic adjustments; enhancing the accumulation of protective proteins such as late embryogenesis abundance (LEA) to prevent cell damage; and so on (Nadeem et al., 2019; Waititu et al., 2021). In the ABA-independent pathway, the major regulators are the CBF/DREB TFs, which belong to the ERF/AP2 family. CBF/DREB TFs activate the expression of a series of downstream non-ABA response stress-responsive genes through binding to the conserved DRE (dehydration-responsive element)/CRT (C-RepeaT) DNA-binding motif in the promoter of their target genes and enhance plant drought resistance (Shinozaki and Yamaguchi-Shinozaki, 2007; Roychoudhury et al., 2013; Fu et al., 2016; Huang et al., 2021). In addition, some non-ABA response NAC and bZIP TFs also play crucial roles in plant drought resistance, which have parallel functions with CBF/DREB TFs (Shinozaki and Yamaguchi-Shinozaki, 2007; Roychoudhury et al., 2013; Fu et al., 2016; Huang et al., 2021). Although the drought resistance mechanisms among different plants are conserved to a certain degree, the drought resistance ability of specific plants strongly depends on the genotype. Under drought stress, the gene responses between drought-tolerant and sensitive genotypes are largely different, and there also exist genotypic-specific responses (Dal Santo et al., 2016; Rocheta et al., 2016; Pieczynski et al., 2018; Sprenger et al., 2018; You et al., 2019; Tarun et al., 2020; Waititu et al., 2021). Therefore, it is of great significance to analyze the transcriptome differences between plant drought-tolerant and drought-sensitive genotypes under drought and uncover the excellent drought resistance genes from the drought-tolerant genotypes for developing drought-resistant crop varieties through gene manipulation.

Tartary buckwheat (*Fagopyrum tataricum* Gaertn.) is a vital medicinal and edible minor grain crop which belongs to the eudicot Polygonaceae family (Li et al., 2019a; Li et al., 2022). It mainly grows in the mountainous areas of western China and the Himalayas and several other regions including Europe and North America (Zhang et al., 2017). China is the largest producing and consuming country of Tartary buckwheat in the world (Zhang et al., 2021a). Tartary buckwheat plays important roles in food security, economic development, and people’s health in China, especially in the western mountainous areas (Li et al., 2022). However, the frequent drought events, which occurred recently in the mountainous areas of western China, especially in autumn, have seriously affected Tartary buckwheat production and threatened the food security in these areas. Thus, it is urgent to explore the wide-transcriptome response of Tartary buckwheat to drought stress and uncover the excellent drought-resistant genes, which will help us breed drought-resistant Tartary buckwheat varieties through molecular manipulation. To date, only one study has investigated the transcriptome response of one Tartary buckwheat genotype to drought stress, which was simulated by 20% polyethylene glycol (PEG-6000) (Huang et al., 2021). Recently, several studies have suggested that the drought resistance of Tartary buckwheat was strongly dependent on genotype (Lu et al., 2017; Lu et al., 2018; Wan et al., 2021). Furthermore, drought stress induced by PEG treatment could not be completely equal to the field soil drought stress. Consequently, it will be more meaningful to compare and analyze the transcriptome difference between Tartary buckwheat drought-tolerant and drought-sensitive genotypes under field soil drought stress, which will help us identify the excellent drought-resistant genes in the drought-tolerant genotype.

To the best of our knowledge, there is no information available about the comparative transcriptome analysis of drought-tolerant and drought-sensitive genotypes of Tartary buckwheat under field soil drought stress. In addition, drought stress, which happened in the reproductive stage, has the most negative effect on crop production. Therefore, in the present study, we performed comparative physiological and transcriptome analyses of drought-tolerant (XZSN) and drought-sensitive (LK3) genotypes under field soil drought stress in the reproductive stage. The aims of this study were to gain insights into the differences in the physiological and molecular mechanisms between drought-tolerant and drought-sensitive genotypes to cope with field soil drought stress and to identify the potentially excellent drought-resistant genes in the drought-tolerant genotypes. Our results provide bases for a better understanding of the genotype-dependent drought resistance response of Tartary buckwheat and potential candidate genes for further Tartary buckwheat drought resistance studies.

## Materials and methods

### Plant materials and stress treatment

The drought-tolerant (XZSN) and drought-sensitive (LK3) genotypes of Tartary buckwheat were used in this study. Seeds of the two genotypes were obtained from the Research Center of Buckwheat Industry Technology of Guizhou Normal University (Guiyang, Guizhou, China). Plants were sown in plastic pots (20-cm depth and 25-cm diameter) containing field soil mixed with an appropriate amount of compound fertilizer. The drought treatment was performed when plants were at the reproductive stage. Before the drought treatment, the plants of XZSN and LK3 were first poured with sufficient water and then subjected to natural drought stress conditions by withholding water for 3, 5, and 7 days. In addition, the 2-day rewatering treatment was also carried out at 8 days of drought treatment. Leaf samples were harvested between 9:30 and 10:00 a.m. from four different plants for each genotype at 0 (before stress), 3, 5, and 7 days and 2 days after rewatering, respectively. All samples from each time point were collected with three biological replicates. For each plant, the fourth, fifth, and sixth leaves from the plant top were collected. For transcriptome analysis, the leaf samples were immediately frozen in liquid nitrogen and stored at −80°C. For the determination of physiological indices, the harvested leaf samples were put on ice and were promptly used to investigate the relative physiological indices.

### Phenotypic and physiological characterizations

The plant phenotype of each genotype was recorded by photographing at each time point. For the determination of physiological indices, the relative water content (RWC) and relative electrolyte leakage (REL) were performed as described in Waititu et al. (2021). The proline content (PC) and soluble sugar content (SSC) were determined according to Dien et al. (2019).

### Total RNA extraction, library construction, and sequencing

Total RNA extraction from each leaf sample was performed using the RNAprep Pure Plant Plus Kit (Tiangen, Beijing, China). The concentration, quality, and integrity of the total RNA were monitored using the NanoDrop spectrophotometer 2000 (NanoDrop, Wilmington, DE, USA) and 1.2% agarose gel electrophoresis. Then, the mRNA from total RNA was purified using the Dynabeads mRNA Purification Kit (Invitrogen, Carlsbad, CA, USA). Purified mRNA was further fragmented as 200–300 bp by divalent cations under elevated temperature in an Illumina proprietary fragmentation buffer and reverse-transcribed into first-strand cDNAs with random hexamer primers and SuperScript™ II (Invitrogen, Carlsbad, CA, USA). Subsequently, the double-stranded cDNAs were synthesized using the NEBNext Ultra RNA Library Prep Kit (NEB, MA, USA). The synthesized double-stranded cDNAs were purified, adenylated at the 3' ends, and ligated to adaptors. The obtained double-stranded cDNAs with adaptors were further enriched by PCR to construct the final sequencing cDNA library. The established cDNA library was sequenced on NovaSeq 6000 platform (Illumina) by Personal Biotechnology Co., Ltd. (Shanghai, China).

### RNA-seq data analysis

The raw data were obtained and the sequence quality was assessed by using FastQC. The adaptor sequences, primers, and low-quality reads were filtered out using Cutadapt (v1.15) software to get the clean reads. After filtering, the clean reads were mapped to the reference genome (http://www.mbkbase.org/Pinku1/) (Zhang et al., 2017) using HISAT2 v2.0.5 (Kim et al., 2013). The aligned reads were assembled into transcripts, and the assembled transcripts from all samples were merged using Cufflinks (Waititu et al., 2021). The obtained unique transcripts were assigned to the annotated reference genes by aligning. The gene expression values were calculated using HTSeq-count (v.0.9.1) (Anders et al., 2015) and normalized to fragments per kilobase of transcript per million fragments mapped (FPKM). Differentially expressed genes (DEGs) were identified by using the software tximport and DESeq2 (Wang et al., 2009). For the identification of DEGs, the |log2(fold change)| of ≥1 and the adjusted *p*-value ≤0.05 were applied.

### Gene ontology and the kyoto encyclopedia of genes and genomes pathway enrichment analysis of DEGs

The gene ontology (GO) enrichment analysis of DEGs was performed by using the software GOseq R package. GO terms with a *p*-value ≤0.05 were defined as significantly enriched GO terms and further divided into the categories of biological process (BP), molecular function (MF), and cellular component (CC). For the Kyoto Encyclopedia of Genes and Genomes (KEGG) pathway enrichment analysis, the DEGs were first mapped to the KEGG pathway term in the KEGG database (Kanehisa et al., 2008). Then, KOBAS 3.0 (Xie et al., 2011) was used to obtain the KEGG enrichment results. KEGG pathway terms with a *p*-value ≤0.05 were assigned as significantly enriched KEGG pathways.

### Weighted gene co-expression network analysis and gene network visualization

The potential drought-resistant genes of the drought-tolerant genotype (XZSN) were used to perform weighted gene co-expression network analysis (WGCNA) to identify the core drought-resistant genes especially TFs based on the previous description (Langfelder and Horvath, 2008). Then, the transcriptional regulatory network among these core drought-resistant TFs and downstream drought-resistant genes was conducted as previously described (Wang et al., 2022). In brief, the position frequency matrices (PFMs) of TFs were downloaded from the PlantTFDB database (Tian et al., 2020); then, the FIMO in the MEME database was used to predict the cis-motif information in the promoter region of these candidate genes (2,000 bp upstream of the initiation codon) under the condition *p <*1e−5 (Grant et al., 2011); finally, the transcriptional regulatory network was constructed by integrating the availability of cis-element binding sites present in the promoter regions of candidate genes and the Pearson correlation coefficient (*r* > 0.8) between these candidate genes and TFs. The network was visualized by Cytoscape (v3.9.1) (Kohl et al., 2011).

### Quantitative real-time polymerase chain reaction analysis

Ten candidate core drought-resistant genes were randomly selected to verify the reliability of the RNA-seq data by quantitative real-time polymerase chain reaction (qRT-PCR). The Tartary buckwheat helicase gene (*HLK/FtPinG0000667700.01*) was used as the internal reference to normalize the expression data. All primer sequences are listed in **Supplementary Table S1**. qRT-PCR was performed as previously described (Li et al., 2021a).

### Statistical analysis

The SPSS software (version 20.0) was used to perform the statistical analysis by one-way analysis of variance (ANOVA) and *t*-test. A *p*-value <0.05 was considered a statistically significant difference.

## Results

### Morphological and physiological differences of XZSN and LK3 plants in response to drought stress

**Figure 1:**
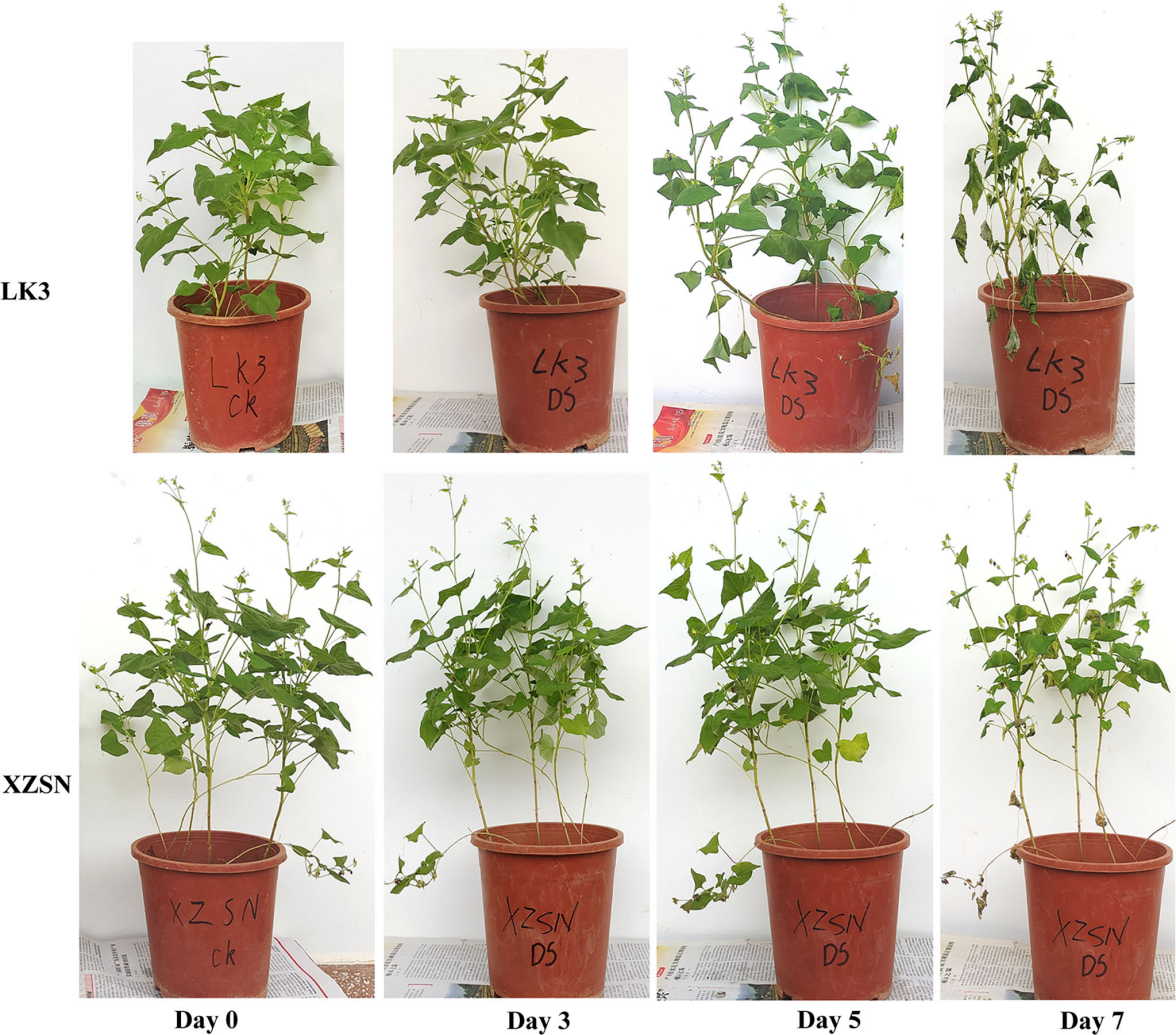
Phenotypic response of the drought-tolerant genotype XZSN and the drought-susceptible genotype LK3. Days 0, 3, 5, and 7 represent natural drought stress conditions by withholding water for 0, 3, 5, and 7 days after pouring sufficient water, respectively.

**Figure 2:**
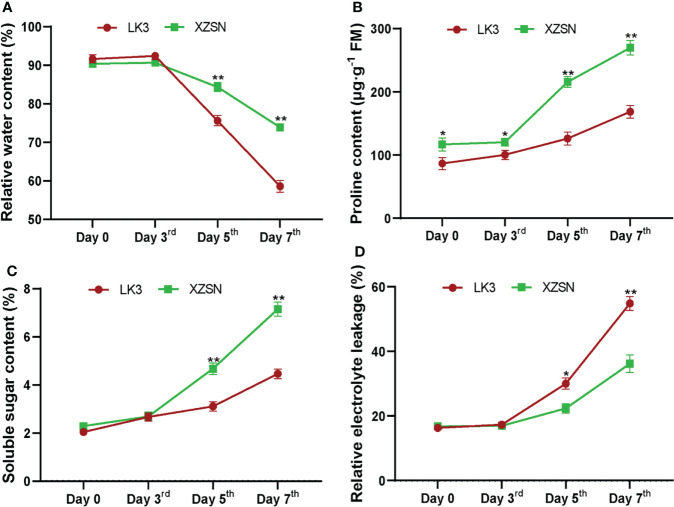
Physiological response of the drought-tolerant genotype XZSN and the drought-susceptible genotype LK3. **(A)** Relative water content (%), **(B)** proline content (μg·g^−1^ FM), **(C)** soluble sugar content (%), and **(D)** relative electrolyte leakage (%). Days 0, 3, 5, and 7 represent natural drought stress conditions by withholding water for 0, 3, 5, and 7 days after pouring sufficient water, respectively. Bars with one (*) and two (**) stars are significantly different at *p <*0.05 and *p <*0.01.

The morphological and physiological differences of XZSN and LK3 plants were investigated at 0, 3, 5, and 7 days under withholding water treatment. As shown in **Figure 1**, there was no visible phenotypic difference observed between the two genotypes at 0 and 3 days of treatment. However, the phenotypes between the two genotypes showed visible differences at 5 days of treatment (**Figure 1**). The leaves of the LK3 plants were rolled and wilted to some degree, while this finding was not observed in the XZSN plants. The rolling and wilting of the leaves of LK3 became more serious at 7 days of treatment. In comparison, the leaves of the XZSN seedlings only displayed slight wilting although some old leaves were dried (**Figure 1**). Consistent with the phenotypic results, no significant difference was observed in the physiological characterizations including RWC, SSC, and REL between LK3 and XZSN at 0 and 3 days of treatment (**Figure 2**). However, the XZSN had significantly higher RWC, PC, and SSC and lower REL than LK3 at 5 and 7 days of treatment (**Figure 2**).

### Transcriptome analysis of XZSN and LK3 plants in response to drought stress

To gain insights into the molecular mechanisms involved in the drought stress resistance of XZSN, we performed RNA-seq for the collected leaf samples of XZSN and LK3 at 0, 3, 5, and 7 days of drought treatment as well as 2 days of rehydration. In total, 30 samples were subjected to RNA-seq, resulting in a total of 13.40 billion raw reads (**Supplementary Table S2**). After removing the adaptor sequences and low-quality sequences, a total of 12.18 billion clean reads were obtained with the samples ranging from 36.42 to 45.61 million. The Q30 base percentage changed from 90.83% to 93.91%, and the GC content varied from 45.47% to 48.81%. Among all the samples, 92.20% to 96.01% of the clean reads were mapped to the reference genome. The Pearson correlation coefficient (PCC) among the biological replicates was over 0.93 for each sample except LK0_1 and XZ0_1, which were deleted from further analysis (**Supplementary Figure S1**). All the results indicated that the RNA-seq was of high quality.

The normalized expression level (FPKM) of each gene was obtained, and the genes with average FPKM ≥1 at least in one tissue sample were considered to be expressed. In total, 18,258 expressed genes, including 17,846 in XZSN and 17,790 in LK3, were identified (**Supplementary Figure S2A**). The number of expressed genes ranged from 15,821 (LKRW) to 16,577 (LK3) for LK3 and 15,743 (XZ5) to 16,629 (XZ3) for XZSN (**Supplementary Figure S2A**), respectively. Among these genes, about 8.22%–9.50%, 9.36%–12.01%, 41.71%–44.30%, and 34.94%–39.58% of the genes displayed very high (FPKM ≥ 100), high (50 ≤ FPKM < 100), moderate (10 ≤ FPKM < 50), and low (1 ≤ FPKM < 10) expression (**Supplementary Figure S2B**), respectively. Interestingly, the LK7 and LKRW samples had the largest and lowest number of genes which displayed very high and high expression, respectively. Overall, these analyses showed that we obtained sufficient coverage of the transcriptome of the drought treatment leaves of these two contrasting Tartary buckwheat genotypes and could be used for further analysis and identification of drought-resistant genes.

### Transcriptome comparison of XZSN and LK3 revealed the vital time point for drought response difference

**Figure 3:**
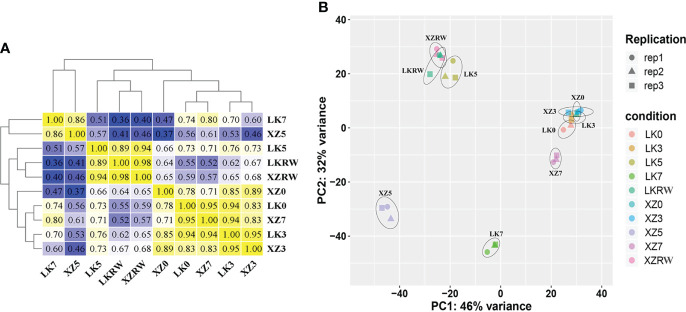
Pearson correlation **(A)** and principal component analyses **(B)** of RNA-seq data from five time points of drought treatment in XZSN and LK3. LK0, LK3, LK5, LK7, and LKRW represent genotype LK3 being subjected to natural drought stress conditions by withholding water for 0, 3, 5, and 7 days and rewatering treatment for 2 days, respectively. XZ0, XZ3, XZ5, XZ7, and XZRW represent genotype XZSN being subjected to natural drought stress conditions by withholding water for 0, 3, 5, and 7 days and rewatering treatment for 2 days, respectively.

To investigate the relationships of leaf transcriptome response to drought stress between XZSN and LK3, hierarchical cluster analysis (HCA) and principal component analysis (PCA) were carried out based on the average FPKM values of the 18,258 expressed genes (**Figure 3**). HCA showed that the samples of the two genotypes at the same treatment time points exhibited higher correlation and clustered except at drought treatment for 5 and 7 days (**Figure 3A**). The PCA analysis displayed LK0 and XZ0, LK3 and XZ3, and LKRW and XZRW being grouped together, while clear separations were observed between LK5 and XZ5 as well as between LK7 and XZ7 (**Figure 3B**). In addition, LK0, LK3, XZ0, and XZ3 were closely clustered together. These indicated that a higher similarity in transcriptional programs and obvious transcriptional differences between the two genotypes existed at drought treatment for 0 and 3 days and at drought treatment for 5 and 7 days, respectively. Furthermore, these also suggested that both genotypes might be subjected to drought stress at drought treatment for 5 and 7 days, and the 5-day drought treatment might be the vital time point to determine the drought resistance difference between the two genotypes at the molecular level.

**Figure 4:**
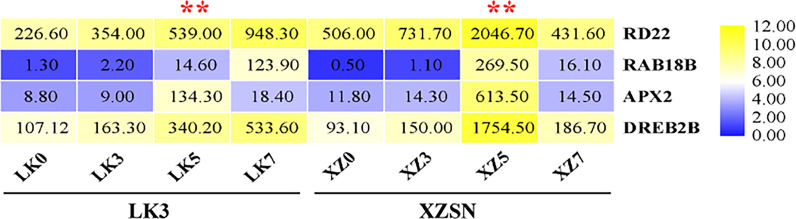
FPKM value of four drought-responsive marker genes in LK3 and XZSN at different treatment points. ** represents the time point of response to drought stress.

To further ascertain that 5 and 7 days of drought treatment as well as the drought treatment for 5 days were the critical time points for the drought transcriptional response difference of the two genotypes subjected to drought stress, the expression levels of *RD22*, *RAB18B*, *APX2*, and *DREB2B*, which are well-known genes of drought response in different plants, were analyzed based on the RNA-seq data. As shown in **Figure 4**, the expression of the four genes was not induced both in XZSN and LK3 at drought treatment for 3 days; however, their expression significantly increased at drought treatment for 5 and 7 days. Interestingly, obviously higher expression levels of the four genes were observed in XZSN than in LK3 at drought treatment for 5 days, although there was no significant expression difference at drought treatment for 0 and 3 days. These indicated that both XZSN and LK3 were really subjected to drought stress at drought treatment for 5 and 7 days and the drought treatment for 5 days was the real key time point that led to the drought resistance difference between the two genotypes at the molecular level.

### Identification of drought-responsive genes in XZSN and LK3

**Figure 5:**
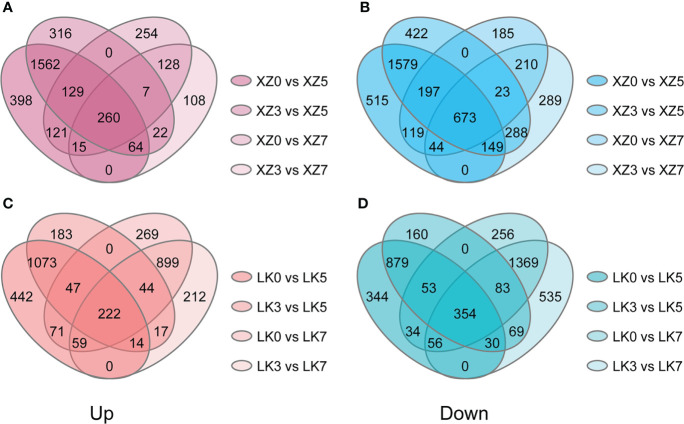
Drought-responsive genes in XZSN and LK3. **(A)** Upregulated drought-responsive genes in XZSN, **(B)** downregulated drought-responsive genes in XZSN, **(C)** upregulated drought-responsive genes in LK3, and **(D)** downregulated drought-responsive genes in LK3.

The physiological and transcriptome data showed that both XZSN and LK3 were subjected to drought stress at drought treatment for 5 and 7 days. Consequently, genes with a significant expression change (|log2(fold change)| of ≥1 and adjusted *p*-value ≤ 0.05) were considered as the drought-responsive genes when they underwent drought treatment for 5 and 7 days compared with genes under drought treatment for 0 and 3 days. As a result, a total of 4,631 (2,015 upregulated and 2,616 downregulated) and 1,360 (410 upregulated and 950 downregulated) genes were identified to be potential drought-responsive genes in XZSN at drought treatment for 5 and 7 days (**Figures 5A, B**), respectively. Similarly, a total of 2,672 (1,356 upregulated and 1,316 downregulated) and 3,086 (1,224 upregulated and 1,862 downregulated) genes were found to have positive and negative responses to drought stress in LK3 at drought treatment for 5 and 7 days (**Figures 5C, D**), respectively. XZSN had more and less drought-responsive genes than LK3 at drought treatment for 5 and 7 days, respectively. In addition, among these potential drought-responsive genes, only 1,306 drought-induced and 1,741 drought-repressed genes overlapped in XZSN and LK3.

### Identification and functional annotation of differentially expressed drought-responsive genes between XZSN and LK3

**Figure 6:**
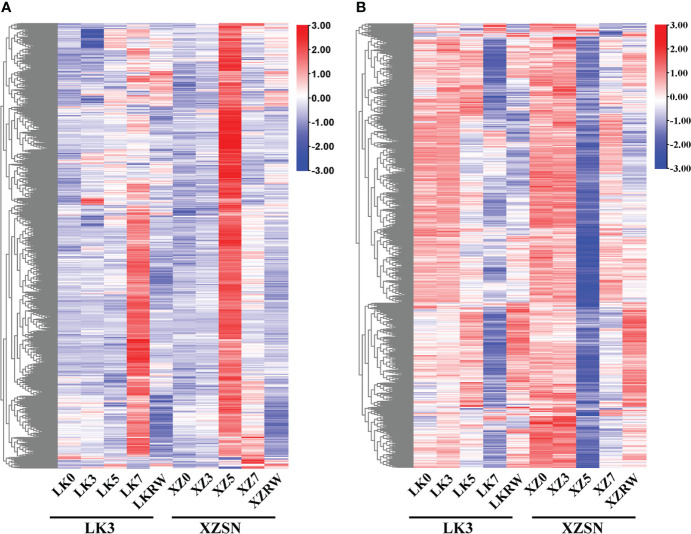
Heatmap of differentially expressed drought-responsive genes between XZSN and LK3. **(A)** Heatmap of upregulated drought-responsive genes in LK3 vs. XZSN. **(B)** Heatmap of downregulated drought-responsive genes in LK3 vs. XZSN.

To gain insights into the potential molecular mechanism of XZSN having a stronger drought resistance ability than LK3 and identify drought-resistant genes in XZSN, the DEGs between ZXSN and LK3 were investigated at drought treatment for 5 and 7 days, respectively. In addition, the DEGs between ZXSN and LK3, which were also the potential drought-responsive genes in XZSN, were considered as the drought-resistant genes that contributed to the higher drought resistance ability of ZXSN. In total, 2,371 (1,168 upregulated and 1,203 downregulated) and 117 (45 upregulated and 72 downregulated) potential drought-responsive genes in XZSN displayed significantly higher and lower expression in XZSN than LK3 at drought treatment for 5 and 7 days, respectively. These genes were identified to be the potential contributors for ZXSN having higher drought resistance ability than LK3. Interestingly, over half of these genes with higher or lower expression in ZXSN than in LK3 at drought treatment for 5 days were obviously upregulated or downregulated in LK3 only at drought treatment for 7 days (**Figure 6**), respectively.

**Figure 7:**
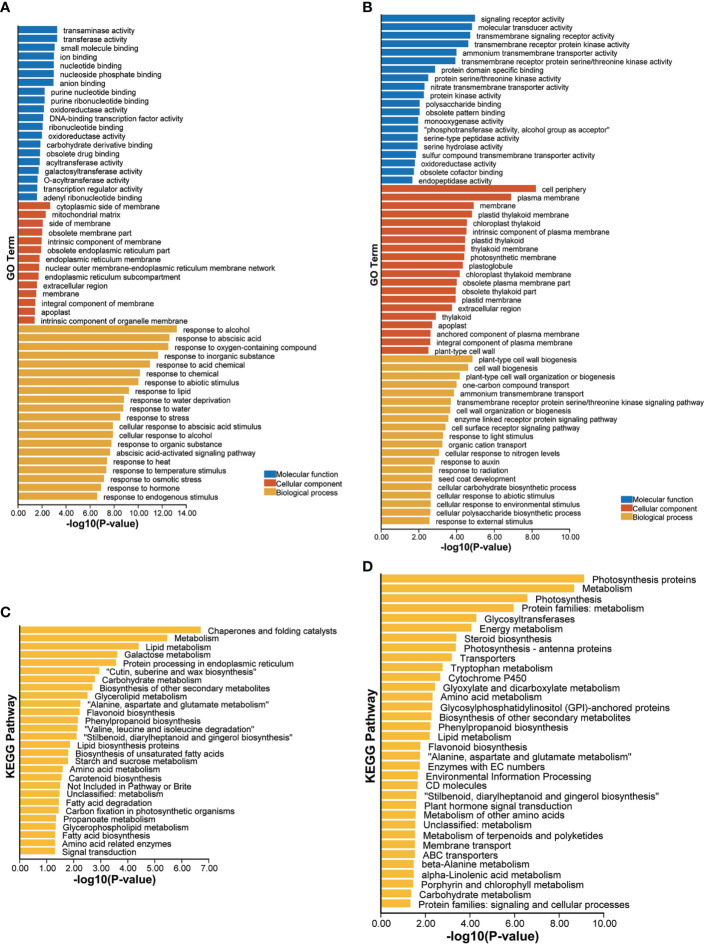
Gene ontology (GO) and Kyoto Encyclopedia of Genes and Genomes **(KEGG)** enrichment analysis of differentially expressed drought-responsive genes between XZSN and LK3. **(A)** GO enrichment of upregulated drought-responsive genes in LK3 vs. XZSN. **(B)** GO enrichment of downregulated drought-responsive genes in LK3 vs. XZSN. **(C)** KEGG enrichment of upregulated drought-responsive genes in LK3 vs. XZSN. **(D)** KEGG enrichment of downregulated drought-responsive genes in LK3 vs. XZSN.

GO enrichment analysis of these upregulated drought-responsive genes of LK3 vs. XZSN obtained 229 significantly enriched GO terms (*p* < 0.05), which could be divided into three functional categories: biological processes (187), cell components (14), and molecular functions (28) (**Supplementary Table S3**). The top 20 GO terms of each functional category are shown in **Figure 7A**. Notably, in the category of biological processes, a large number of genes were enriched in response to stress (abiotic stimulus, water deprivation, heat, cold, osmotic stress, salt stress, oxidative stress, reactive oxygen species, alcohol, hydrogen peroxide, cadmium, and so on), hormone (abscisic acid and gibberellin), carbohydrate and sucrose metabolism (phenylpropanoid metabolic process, glutamine family amino acid metabolic process, inositol phosphate metabolic process, organic acid metabolic process, hexose metabolic process, fatty acid metabolic process, lipid metabolic process, flavonoid metabolic process, and so on), and so on (**Figure 7A**; **Supplementary Table S3**). In contrast, a total of 159 significantly enriched GO terms, consisting of 99 biological process terms, 27 cell component terms, and 33 molecular function terms, were identified for these downregulated drought-responsive genes of LK3 vs. XZSN (**Figure 7B**; **Supplementary Table S3**). In the category of biological processes, these enriched GO terms were primarily involved in cell wall biogenesis, cell wall organization or biogenesis, response to auxin, cellular response to abiotic stimulus, and cellular response to an environmental stimulus (**Figure 7B**; **Supplementary Table S3**). The KEGG pathway enrichment analysis found that the up- and downregulated drought-responsive genes were significantly enriched to 27 and 34 pathways (*p* < 0.05) of LK3 vs. XZSN, respectively (**Figures 7C, D**; **Supplementary Table S4**). For the upregulated drought-responsive genes, the enriched pathways were primarily involved in chaperones and folding catalysts, lipid biosynthesis and metabolism, wax biosynthesis, phenylpropanoid and flavonoid biosynthesis, amino acid biosynthesis and metabolism, starch and sucrose metabolism, fatty acid biosynthesis and degradation, carotenoid biosynthesis, and so on. In contrast, photosynthesis, energy metabolism, steroid biosynthesis, glyoxylate and dicarboxylate metabolism, and plant hormone signal transduction pathways were significantly enriched in the downregulated drought-responsive genes of LK3 vs. XZSN (**Figures 7C, D**; **Supplementary Table S4**).

### Identification and functional annotation of positive core drought-resistant genes in XZSN

**Figure 8:**
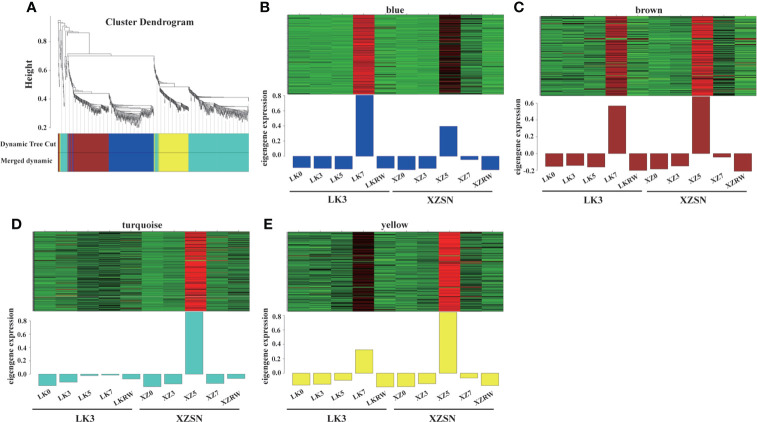
Co-expression network of the 1,206 potentially positive drought-resistant genes in XZSN. **(A)** Hierarchical clustering tree (dendrogram) of all genes. **(B)** Blue module, **(C)** brown module, **(D)** turquoise module, and **(E)** yellow module.

To identify the positive core drought-resistant genes in XZSN, we selected the 1,206 upregulated differentially expressed drought-responsive genes in LK5_vs_XZ5 (1,168) and LK7_vs_XZ7 (45) to further perform the WGCNA analysis. As a result, 851 out of the 1,206 genes were assigned to four modules (blue, brown, turquoise, and yellow) (**Figure 8**). The blue, brown, turquoise, and yellow modules contain 220, 182, 301, and 148 genes, respectively. The genes in the blue, brown, and yellow modules were highly expressed both in ZX5 and LK7, but their expression levels were higher (brown and yellow) and lower (blue) in XZ5 than in LK7, respectively. In contrast, genes from the turquoise module showed a specific high expression in ZX5 (**Figure 8**). Because drought treatment for 5 days was the real key time point to determine the drought resistance difference between XZSN and LK3, the 851 genes were considered as the core drought-resistant genes in XZSN and further used to perform functional annotation.

#### TFs

A total of 67 TFs were identified among the 851 positive core drought-resistant genes in XZSN, consisting of 26, 12, 21, and 8 from the blue, brown, turquoise, and yellow modules (**Supplementary Table S5**), respectively. The 67 TFs could be classified into 21 families (TFs from the same family were named based on their number), and the top 5 largest TF families were NAC (12), MYB (7), HD-ZIP (7), bZIP (6), and WRKY (5) (**Supplementary Table S5**). Among these TFs, two bZIP TF genes (*FtPinG0003196200.01*/*FtbZIP5* and *FtPinG0002143600.01*/*FtbZIP83*), which are homologous to *AtABF2*, have been demonstrated to play a positive regulatory role in drought resistance of transgenic *Arabidopsis* (Li et al., 2019b; Li et al., 2020). Four NAC TF genes (*FtPinG0002561000.01*, *FtPinG0005791100.01*, *FtPinG0006087500.01*, and *FtPinG0005167000.01*) are homologous to *ANAC019*, *ANAC72*/*RD26*, *NAC029*, and *ANAC2*, which are necessary for drought resistance in *Arabidopsis* (Fujita et al., 2011), respectively. In addition, some *Arabidopsis thaliana* homologous genes of the rest of the 61 TFs were involved in biotic and abiotic stress response or resistance, although there was no report about their drought resistance (**Supplementary Table S5**). Notably, among the 67 TFs, only 15 were induced in LK3 at drought treatment for 5 days, but 44 out of the remaining 52 TFs were upregulated in LK3 at drought treatment for 7 days (**Supplementary Table S5**). Furthermore, 8 TFs were specifically induced by drought stress in XZSN (**Supplementary Table S5**).

**Figure 9:**
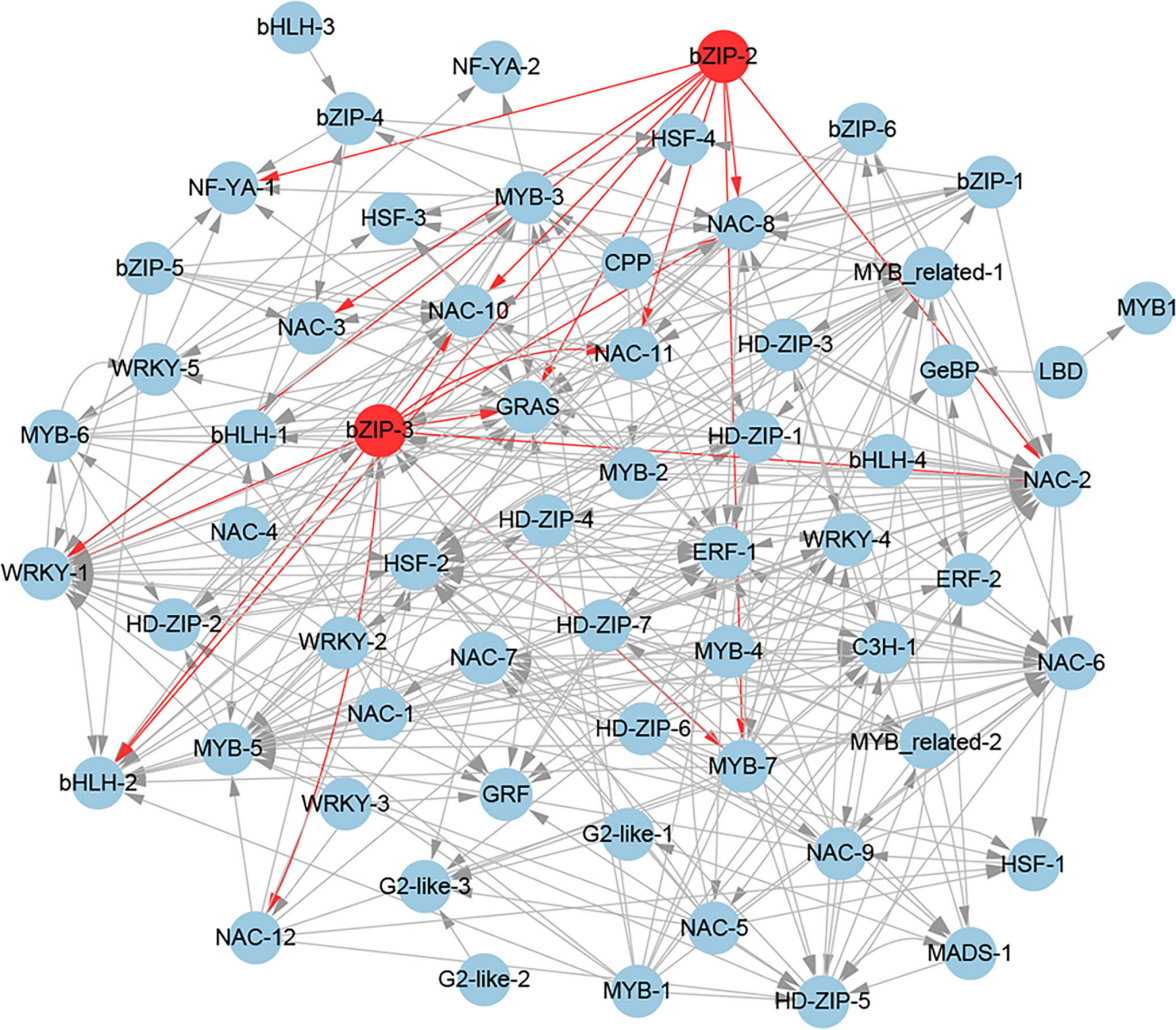
The regulatory network of potential drought-resistant TFs in XZSN. TFs (bZIP-2 and bZIP-3), labeled by a red circle, represents the homologs of key TFs (ABFs) of the abscisic acid (ABA) signal pathway in *Arabidopsis*.

To further gain insights into the transcriptional regulatory relationship among these TFs, we constructed their potential regulatory network based on the expression correlations (*r* > 0.8) and the potential binding sites of these TF promoters (**Figure 9**). The regulatory network consists of 62 TFs. In this regulatory network, 11 potential target genes (*bHLH-2*, *GRAS*, *MYB-7*, *NAC-2*, *NAC-3*, *NAC-8*, *NAC-10*, *NAC-11*, *NAC-12*, *NF-YA-1*, and *WRKY-1*) of the homologous genes (*bZIP-2* and *bZIP-3*) of *AtABFs* were found. Notably, among the 11 potential target genes, the *Arabidopsis* homologous genes of *NAC-3*, *NAC-8*, and *NAC-10* were the direct target genes of *AtABFs* in drought resistance (Hickman et al., 2013). In addition, *bHLH-2*, *C3H-1*, *ERF-1*, *GRAS*, *HSF-2*, *MYB-3*, *MYB-5*, *NAC-2*, *NAC-6*, and *WRKY-1* had the largest number of potential upstream regulatory TFs (**Figure 9**).

### Genes involved in ABA biosynthesis, signal transduction, and response

**Figure 10:**
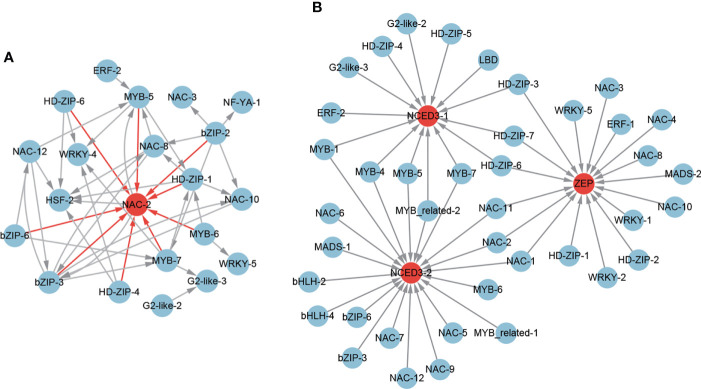
The regulatory network of ABA-responsive TFs **(A)** and ABA biosynthesis genes **(B)** from the core drought-resistant genes in XZSN.

Among the 851 positive core drought-resistant genes in XZSN, 3 ABA biosynthesis genes (1 *ZEP*/*ABA1* and 2 *NCED3*) and 8 ABA signal transduction genes (1 *PYL*/*PYR*, 4 *PP2C*, 1 *SnRK2.6*/*OST1*, and 2 *AREB*/*ABF*) were found (**Supplementary Table S6**). In addition, according to the GO and *Arabidopsis* homologous annotation, 91 out of the 851 core drought-resistant genes in XZSN were identified to be ABA-responsive genes (**Supplementary Table S6**). Among the 91 ABA-responsive genes, 23 belonged to TFs including 1 bHLH, 1 bZIP, 1 ERF, 2 HSF, 2 G2-like, 4 HD-ZIP, 4 MYB, 5 NAC, 2 NF-YA, and 1 WRKY. The potential transcriptional regulatory network among these ABA-responsive TFs is shown in **Figure 10A**. The regulatory network consisted of 21 TFs, and *NAC-2* had the largest number of putative upstream regulators. For the rest of the 68 ABA-responsive genes, most of them were further identified to be stress-responsive genes, which were primarily involved in drought, salt, cold, heat, and oxidative stress responses (**Supplementary Table S6**). Notably, 2 ABA biosynthesis genes (1 *ZEP*/*ABA1* and 1 *NCED3*) were not induced in LK3 at drought treatment for 5 days (**Supplementary Table S6**). Coincidentally, 5 ABA signal transduction genes (3 *PP2C*, 1 *SnRK2.6*/*OST1*, and 1 *AREB*/*ABF*) and 63 ABA-responsive genes were also not induced in LK3 at drought treatment for 5 days (**Supplementary Table S6**). However, all identified ABA biosynthesis and ABA signal transduction genes as well as most ABA-responsive genes were obviously upregulated in LK3 at drought treatment for 7 days (**Supplementary Table S6**).

To further identify the potential TFs that regulated the ABA biosynthesis, the expression correlations (*r* > 0.8) among 67 potential drought-resistant TFs and 3 ABA biosynthesis genes as well as the potential binding sites of the 3 ABA biosynthesis gene promoters were analyzed. A total of 17 (1 ERF, 5 HD-ZIP, 1 MADS, 7 NAC, and 3 WRKY), 14 (1 ERF, 2 G2-like, 5 HD-ZIP, 1 LBD, 1 MYB-related, and 4 MYB), and 20 (2 bHLH, 2bZIP, 1 MADS, 2 MYB-related, 5 MYB, and 8 NAC) TFs were identified to be the putative regulators of the ABA biosynthesis genes *ZEP*, *NCED3-1*, and *NCED3-1* (**Figure 10B**), respectively. Among these TFs, 3 (HD-ZIP-3, HD-ZIP-6, and HD-ZIP-7), 3 (NAC-1, NAC-2, and NAC-3), and 4 (MYB-4, MYB-5, MYB-7, and MYB-related-2) TFs were found as the common regulators of *ZEP* and *NCED3-1*, *ZEP* and *NCED3-2*, and *NCED3-1* and *NCED3-2*, respectively. Notably, among these TFs, 18 were also ABA-responsive TFs (**Supplementary Table S6**).

### Genes involved in non-ABA signal molecules

Among the 851 positive core drought-resistant genes in XZSN, we found 3 non-ABA signal molecules biosynthesis genes, namely, 1 carbon monoxide (CO) biosynthesis gene (*HO1*, heme oxygenase), 1 hydrogen sulfide (H_2_S) biosynthesis gene (*OAS-TL*, cytosolic O-acetylserine(thiol)lyase), and 1 melatonin biosynthesis gene (*COMT1*, caffeic acid/5-hydroxyferulic acid O-methyltransferase) (**Supplementary Table S7**). Notably, the *HO1* and *OAS-TL*, involved in CO and H_2_S biosynthesis, were specifically induced in XZSN at drought treatment for 5 days. In contrast, the melatonin biosynthesis gene *COMT1* was upregulated in LK3 at drought treatment for 7 days, although it was not induced in LK3 at drought treatment for 5 days (**Supplementary Table S7**).

### Genes involved in stomatal closure and cuticular wax biosynthesis

A total of 7 ABA-dependent stomatal closure genes were found among the 851 positive core drought-resistant genes in XZSN (**Supplementary Table S8**), consisting of 2 ABA transporter genes which are homologous to *AtABCG22* and *OsABCG5* with the function of transporting ABA into the leaf guard cell to regulate stomatal closure (Kuromori et al., 2011; Matsuda et al., 2016), 1 NADPH/respiratory burst oxidase gene (*RBOHF*), 1 anion transporter gene (*ALMT12*), 2 nucleocytoplasmic lectin genes (*EULS3*), and 1 receptor-like cytoplasmic kinase gene (*CDL1*). Furthermore, 1 ABA-independent stomatal closure gene (hexokinase, *HXK1*) was also identified (**Supplementary Table S8**). Among these 8 stomatal closure genes, only 1 was induced by drought treatment for 5 days in LK3, but 5 of them were upregulated in LK3 under drought treatment for 7 days (**Supplementary Table S8**). For cuticular wax biosynthesis, a total of 10 homologous genes involved in cuticular wax biosynthesis were identified in the 851 positive core drought-resistant genes in XZSN (**Supplementary Table S8**). These genes included 1 long-chain acyl-CoA synthetase (*LACS1*), 2 β-ketoacyl-coenzyme A synthases (*KCS1* and *KCS6*), 1 β-hydroxyacylcoenzyme A dehydratase (*HCD*/*PAS2*), 2 BAHD acyltransferase (*CER1*), 2 sterol desaturase (*CER3*/*WAX2*), 1 alcohol-forming fatty acyl-CoA reductase (*CER4*/*FAR3*), and 1 wax ester synthase and diacylglycerol acyltransferase (*WSD1*) (**Supplementary Table S8**). In addition, 2 homologs to GPI-anchored lipid transfer protein (2 *LTPG2*) were also identified, involved in cuticular wax transport. Notably, among the 10 cuticular wax biosynthesis and 2 wax transport genes, only 1 was upregulated in LK3 at drought treatment for 5 days, but 11 genes were induced in LK3 at drought treatment for 7 days (**Supplementary Table S8**).

**Figure 11:**
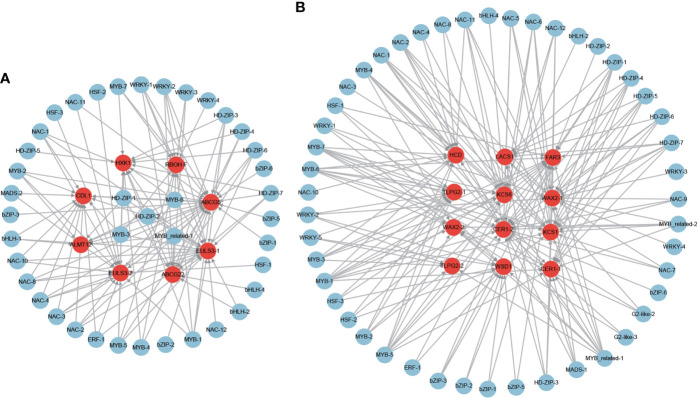
The regulatory network of stomatal closure **(A)** and cuticular wax biosynthesis and transport **(B)** genes from the core drought-resistant genes in XZSN.

To further identify the potential regulator of these stomatal closure and wax biosynthesis and transport genes, the putative regulatory network was constructed (**Figure 11**). A total of 41 and 47 TFs were identified to be the potential regulators of the stomatal closure and wax biosynthesis and transport genes (**Figures 11A, B**), respectively. Among the TFs, some were predicted as the regulators of both stomatal closure and wax biosynthesis genes. Furthermore, some played the putative regulatory role in multiple stomatal closure or wax biosynthesis and transport genes. For example, MYB-3, MYB-6, MYB_related-1, HD-ZIP-1, and HD-ZIP-2 were the potential regulators of 7, 7, 6, 6, and 6 stomatal closure genes (**Figure 11A**), respectively. MYB-1, MYB-5, MYB-6, and MYB-7 were the putative regulators of 8, 8, 7, and 7 wax biosynthesis and transport genes (**Figure 11B**), respectively. Notably, MYB-6, which is the ABA-responsive TF, was predicted as the regulator of 7 stomatal closure and 7 wax biosynthesis and transport genes.

### Genes involved in reactive oxygen species scavenging

**Figure 12:**
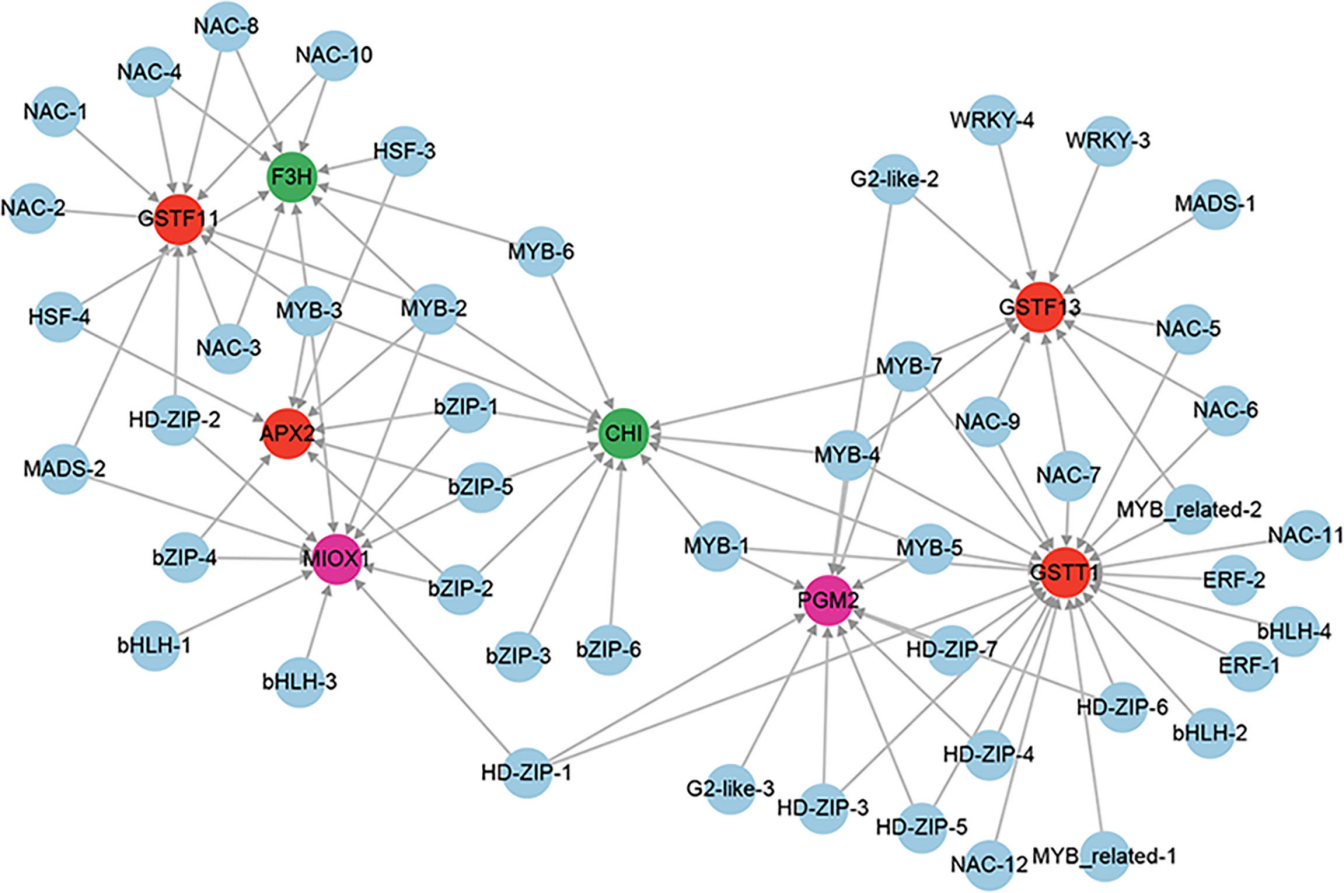
The regulatory network of ROS scavenging genes from the core drought-resistant genes in XZSN. Genes labeled by a red circle encode the enzymatic antioxidants. Genes labeled by green and magenta circles represent the genes involved in non-enzymatic antioxidant biosynthesis.

A total of 6 genes involved in enzymatic antioxidants from the 851 positive core drought-resistant genes in XZSN were identified, including 1 ascorbate peroxidase (*APX*), 2 peroxidase (*PRXs*), and 3 glutathione S-transferase (*GSTFs*) (**Supplementary Table S9**). In addition, 2 (*PGM2* and *MIOX1*) and 2 (*CHI* and *F3H*) genes, involved in the biosynthesis of the non-enzymatic antioxidant ascorbic acid and flavonoids, were also identified (**Supplementary Table S9**), respectively. Only 3 out of the 10 genes were induced in LK3 at drought treatment for 5 days. Furthermore, 4 genes, which were not induced in LK3 at drought treatment for 5 days, were upregulated in LK3 at drought treatment for 7 days (**Supplementary Table S9**). The putative regulators of *APX*, *GSTFs*, *PGM2*, *MIOX1*, *CHI*, and *F3H* were identified as well as the potential regulatory network as shown in **Figure 12**.

### Genes involved in osmotic adjustment

**Figure 13:**
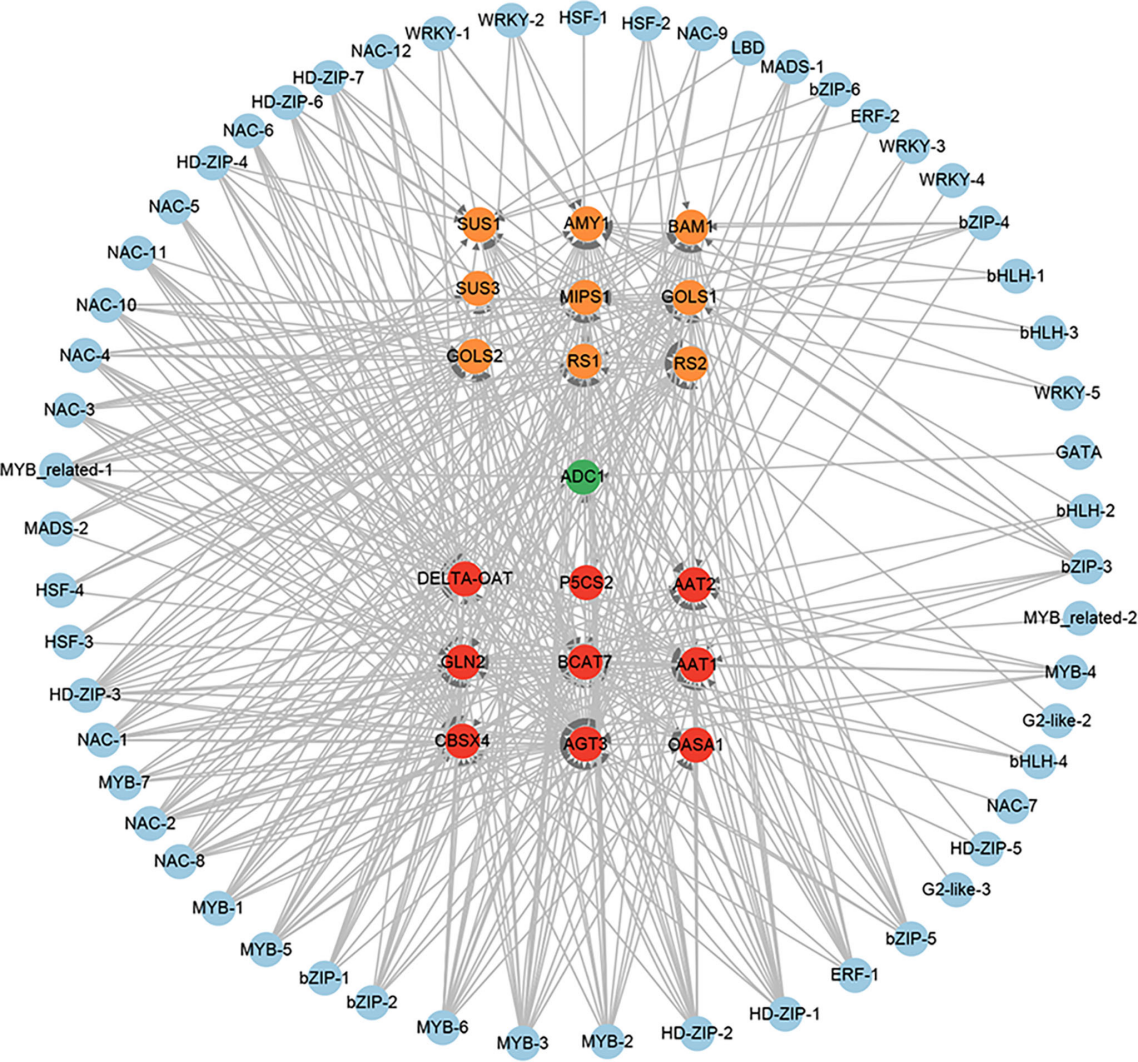
The regulatory network of osmotic adjustment genes from the core drought-resistant genes in XZSN. Genes labeled by yellow, green, and red circles represent the soluble sugar, amino acid, and polyamine biosynthesis genes, respectively.

A total of 12 amino acid biosynthesis genes were identified among the 851 positive core drought-resistant genes in XZSN. These genes encoded the key enzymes involved in glutamine (1 glutamine synthetase), glutamate (1 glutamate synthase), aspartate (2 aspartate aminotransferase), glycine (2 alanine-glyoxylate aminotransferase), cysteine (1 O-acetylserine(thiol)lyase), methionine (1 cystathionine β-lyase), and proline (1 ornithine delta-aminotransferase and 1 pyrroline-5-carboxylate synthetase) biosynthesis, as well as in valine, leucine, and isoleucine (1 branched-chain amino acid transaminase) biosynthesis. One gene encoding the rate-limiting enzyme (arginine decarboxylase, *ADC1*) of polyamine biosynthesis was also found (**Supplementary Table S10**). In addition, 9 genes involved in soluble sugar biosynthesis were identified, which contained 2, 5, and 2 for sucrose biosynthesis, raffinose biosynthesis, and starch degradation (**Supplementary Table S10**), respectively. Among these identified genes, most of the genes involved in amino acid biosynthesis and starch degradation were not induced in LK3 at drought treatment for 5 days, but most of them were upregulated at drought treatment for 7 days. In contrast, genes involved in polyamine and raffinose biosynthesis were induced in LK3 at both drought treatments for 5 and 7 days (**Supplementary Table S10**). The putative regulatory network of amino acid, polyamine, sucrose, and raffinose biosynthesis genes was constructed and shown in **Figure 13**. A total of 49, 6, and 44 TFs were predicted as the potential regulators of amino acid, polyamine, and sucrose and raffinose biosynthesis genes, respectively. Among these TFs, some played potential regulatory roles in two or more osmotic adjustment solutes (**Figure 13**). Notably, among these TFs, MYB-3, MYB-6, HD-ZIP-1, HD-ZIP-2, HD-ZIP-3, MYB-2, MYB_related-1, and NAC-1 were predicted as the regulators of 17, 16, 16, 16, 14, 13, and 12 out of the 21 osmotic adjustment genes, respectively.

### Genes involved in cell damage prevention

A total of 3 late embryogenesis abundant (*LEA*) and 5 heat shock protein (*HSP*) genes were identified among the 851 positive core drought-resistant genes in XZSN (**Supplementary Table S11**), which play crucial roles in preventing cell damage under different abiotic stresses (Priya et al., 2019; Shi et al., 2020). Half of these genes were not upregulated in LK3 at drought treatment for 5 days, while 2 of them were induced in LK3 at drought treatment for 7 days. In addition, 2 *HSP* genes were specifically induced in XZSN at drought treatment for 5 days (**Supplementary Table S11**).

### Identification of the key downstream TFs involved in drought resistance in tartary buckwheat

**Figure 14:**
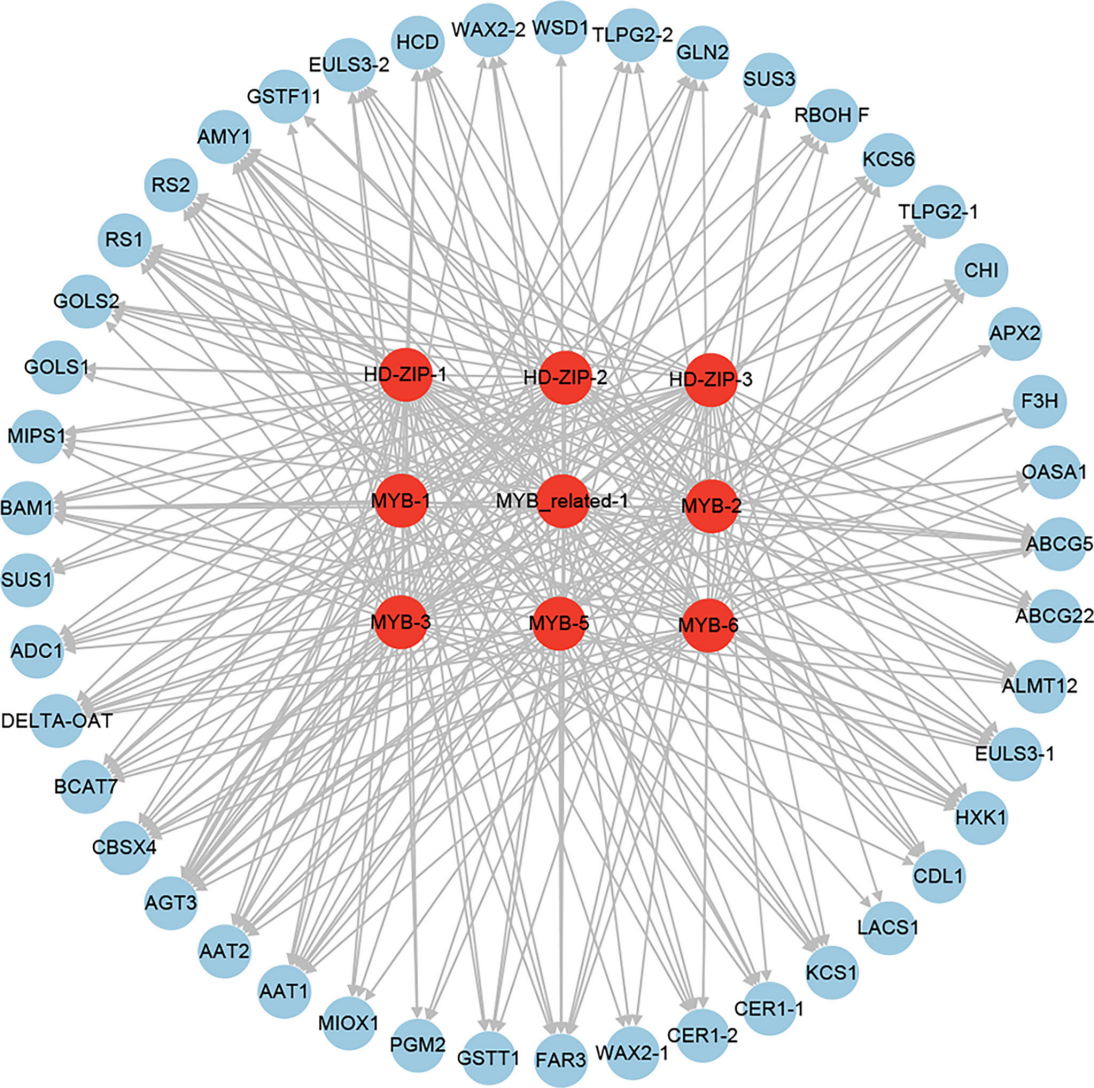
The regulatory network of the key downstream TFs involved in drought resistance in Tartary buckwheat.

To identify the key downstream TFs involved in drought resistance in Tartary buckwheat, we performed an integrated analysis of the above identified TFs, which were putative as the regulators of the expression of 20 water holding (8 stomatal closure and 12 cuticular wax biosynthesis and transport), 7 reactive oxygen species scavenging, and 18 osmotic adjustment (8 amino acid, 1 polyamine, and 9 soluble sugar biosynthesis) genes. As a result, 3 HD-ZIPs (HD-ZIP-1, HD-ZIP-2, and HD-ZIP-3), 1 MYB-related (MYB-related-1), and 5 MYBs (MYB-1, MYB-2, MYB-3, MYB-5, and MYB-6) were found as the putative regulators of 31, 25, 26, 28, 27, 26, 34, 27, and 32 out of the 45 drought-resistant functional genes (**Figure 14**). This indicated that HD-ZIP and MYB TFs might be the key downstream TFs of drought resistance in Tartary buckwheat.

### Confirmation of the core droughtresistant genes by qRT-PCR

**Figure 15:**
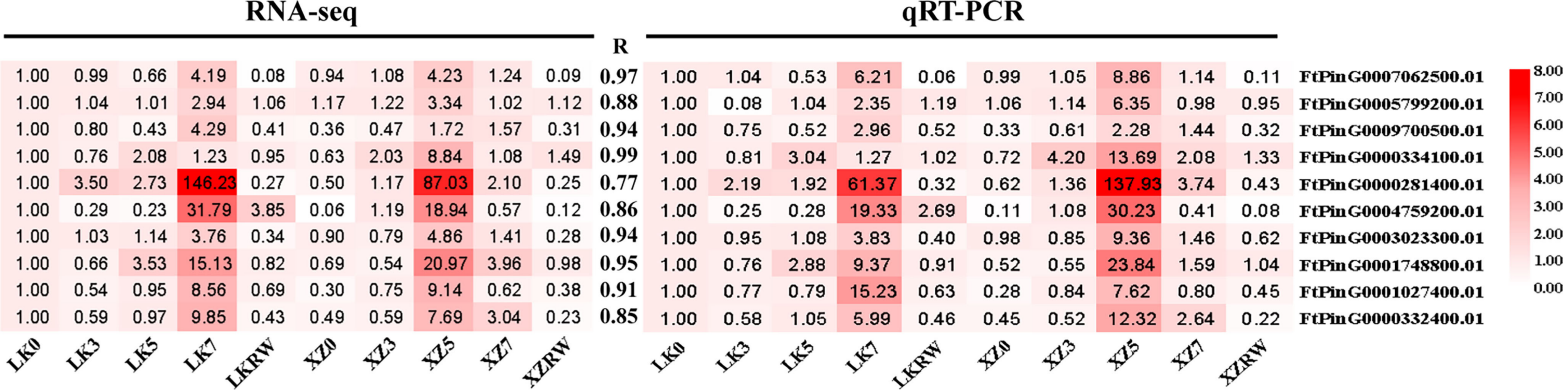
Correlation analysis between RNA-seq and qRT-PCR methods. The expression fold change of genes is used to construct the heatmaps. The LK0 is used as a control. The left heatmap represents the RNA-seq, and the right heatmap represents qRT-PCR.

To confirm the reliability of these identified core drought-resistant genes from XZSN, 10 TFs from the 851 core drought-resistant genes were selected to perform the qRT-PCR analysis in the leaf samples of XZSN and LK3 with treatment by naturally withholding water for 0, 3, 5, and 7 days and rewatering for 2 days. As shown in **Figure 15**, a highly significant correlation (*R* ≥ 0.77) was observed between qRT-PCR (log2 fold change) and RNA-seq (log2 fold change) data, thus verifying the authenticity of the identified core drought-resistant genes from XZSN in this study.

## Discussion

### Morphological and physiological responses to drought

In many plants, the morphological and physiological indices highlight that the tolerant and susceptible genotypes respond differently to drought stress (Dossa et al., 2017; Wang et al., 2018b; Kumar et al., 2019; Harb et al., 2020; Ma et al., 2021; Wan et al., 2021; Waititu et al., 2021; Zhao et al., 2021; Zhang et al., 2021a). For example, the RWC of the leaf of the tolerant genotype was higher and the wilting was slower compared with the susceptible genotype. In addition, the accumulations of some protective substances such as proline, polyamine, soluble sugar, soluble protein, and so on were faster and higher in the tolerant genotype than in the susceptible genotype. In contrast, the malondialdehyde (MAD) content and REL, which indirectly reflect the degree of cell damage, were lower in the tolerant genotype than in the susceptible genotype. Consistent with these observations in other plants, our study found that the leaves of the Tartary buckwheat drought-susceptible genotype LK3 rolled and wilted quicker and more serious than the drought-tolerant genotype XZSN under drought stress (**Figure 1**). Physiological characterization analyses showed that the RWC, PC, and SSC in the drought-tolerant genotype XZSN were significantly higher than in the drought-susceptible genotype LK3, while the REL showed the opposite result (**Figure 2**). These indicated that the XZSN genotype was more effective in resisting drought stress than the LK3 genotype by reducing the leaf water loss, more quickly accumulating protective substances, and reducing cell membrane damage.

### Transcriptional differences between drought-tolerant and drought-susceptible genotypes of tartary buckwheat for drought stress response

Integrating the physiological characterizations and the expression of the drought-responsive marker genes, our results showed that both the drought-tolerant (XZSN) and the drought-susceptible (LK3) genotypes were subjected to drought stress at nature drought treatment for 5 and 7 days. The analysis of the drought stress-responsive genes in XZSN and LK3 found that the XZSN had more drought stress-responsive genes than LK3 in the early stage (drought treatment for 5 days) of drought stress. However, in the later stage (drought treatment for 7 days) of drought stress, the situation was just the opposite. In addition, only about half of these drought stress-responsive genes overlapped between XZSN and LK3. These indicated that XZSN and LK3 had the same and different transcriptional responses for drought stress and the transcriptional differences of the early stage of drought stress might be the key reason for leading to the drought resistance ability difference between them.

### Rapid and dramatic transcriptional reprogramming of drought-resistant genes occurs in the drought-tolerant genotype

A total of 1,206 and 1,274 genes were identified to be the potential positive (upregulated in LK3 vs. XZSN) and negative (downregulated in LK3 vs XZSN) contributors for XZSN having higher drought resistance ability than LK3, respectively. GO enrichment analysis showed that a large number of upregulated genes in LK3 vs. XZSN were enriched in response to stress (including water deprivation, heat, cold, osmotic stress, salt stress, oxidative stress, hydrogen peroxide, cadmium, and so on), hormone (abscisic acid and gibberellin), carbohydrate, sucrose, metabolism, and so on (**Figure 7A** **;** **Supplementary Table S3**). In contrast, the downregulated genes in LK3 vs. XZSN were primarily enriched in response to auxin, cellular response to abiotic stimulus, and cellular response to an environmental stimulus (**Figure 7B** **;** **Supplementary Table S3**). These indicated that the identified genes could be the candidate genes for XZSN having higher drought resistance ability than LK3. In some plants, it was shown that the rapid and dramatic transcriptional reprogramming of the stress tolerance genotype contributes to higher stress resistance than the susceptible genotype (Dossa et al., 2017; Simopoulos et al., 2020; Waititu et al., 2021; Yang et al., 2021). Interestingly, among these identified genes, over 90% of the genes were identified at drought treatment for 5 days, and over half of them in LK3 were not induced or inhibited at drought treatment for 5 days but were obviously upregulated or downregulated at drought treatment for 7 days (**Figure 6**). Therefore, our results also indicated that the quick and strongly active transcriptional reprogramming of the drought-resistant genes in XZSN under drought stress could be the major reason for its higher drought resistance ability than LK3.

### Core drought-resistant genes and the potential transcriptional regulatory network in XZSN

The positive regulation of gene expression plays the most important role in plant drought resistance (Hu and Xiong, 2014). Based on the WGCNA analysis of 1,206 positive contributors for XZSN having higher drought resistance ability than LK3, we identified 851 genes to be the core drought-resistant genes in XZSN. According to their homologous function, we classified these core drought-resistant genes and explored their potential transcriptional regulatory network.

#### TFs

TFs are the master regulators of plant drought resistance. Many TFs such as AREB/ABF, AP2/ERF, MYB, NAC, HD-ZF, HD-ZIP, bHLH, C2H2-ZF, B3, WRKY, and NF-Y TFs have been confirmed to play roles in drought resistance in different plant species (Fujita et al., 2011; You et al., 2019; Waititu et al., 2021). In our study, a total of 67 TFs from 21 families were identified in the 851 core drought-resistant genes in XZSN. Among the TF families, some new TF families such as C3H, G2-like, HSF, MYB-related, and MADS were found except for some known TF families involved in plant drought resistance. Notably, among these TFs, two bZIP TFs (FtPinG0003196200.01/FtbZIP5 and FtPinG0002143600.01/FtbZIP83) have been demonstrated to play a positive regulatory role in drought resistance in transgenic *Arabidopsis* (Li et al., 2019b; Li et al., 2020). Four NAC TFs are the homologs of *Arabidopsis* drought-resistant TFs (ANAC019, ANAC72/RD26, NAC029, and ANAC2, respectively) (Fujita et al., 2011). These indicated that these TFs have a conserved function in different plants to resist drought stress. In addition, we also found that the *Arabidopsis* homologs of some identified TFs in this study were involved in biotic and abiotic stress resistance, although there was no report about their drought resistance based on the homologous function annotation. All these suggested that the TFs identified by us might be the master regulators of drought resistance in XZSN, and it was reliable to identify them as the drought-resistant candidate TFs. Notably, most of these TFs were earlier induced by drought stress in XZSN than in LK3. Furthermore, some TFs were specifically induced in XZSN. These indicated that the rapid, strong, and special activation of the expression of drought-resistant TFs in XZSN contributed to its higher drought resistance ability than LK3.

Based on the expression correlation and the potential binding sites of the 67 TFs promoters, we constructed the putative regulatory network of these TFs. In the regulatory network, we found that bZIP-2 and bZIP-3, which are the homologs of *Arabidopsis* AtABFs, had 11 potential target TF genes (*bHLH-2*, *GRAS*, *MYB-7*, *NAC-2*, *NAC-3*, *NAC-8*, *NAC-10*, *NAC-11*, *NAC-12*, *NF-YA-1*, and *WRKY-1*). Notably, among the 11 potential target TF genes, the *Arabidopsis* homologs (*ANAC072*/*RD26*, *ANAC019*, and *ANAC072*/*RD26*) of *NAC-3*, *NAC-8*, and *NAC-10* were the direct target genes of AtABFs in drought resistance (Hickman et al., 2013). In addition, the *Arabidopsis* homologs (*MYB32*, *ANAC2*, *NAC029*, and *ANAC2*) of *MYB-7*, *NAC-2*, *NAC-11*, and *NAC-12* were responsive to drought or salt stress (**Supplementary Table S5**). These suggested that we constructed the drought-resistant TF regulatory network to be credible to some extent, and the 8 TF genes except *NAC-3*, *NAC-8*, and *NAC-10* might be the direct target genes of bZIP-2 and bZIP-3 and played crucial regulatory roles in Tartary buckwheat drought resistance. In addition, we also found that *bHLH-2*, *C3H-1*, *ERF-1*, *GRAS*, *HSF-2*, *MYB-3*, *MYB-5*, *NAC-2*, *NAC-6*, and *WRKY-1* have the largest number of putative upstream regulatory TFs (**Figure 9**). This implied that these TFs might be the crucial downstream regulatory TFs that directly regulated the expression of the functional genes in drought resistance in Tartary buckwheat.

#### ABA signaling

In plants, the ABA content increases rapidly under drought stress and further activates the ABA-dependent drought resistance pathway, which is the major and conservative molecule signal pathway for plant drought resistance (Shinozaki and Yamaguchi-Shinozaki, 2007; Roychoudhury et al., 2013; Saradadevi et al., 2017). Among the 851 core drought-resistant genes in XZSN, 3 ABA biosynthesis genes (1 *ZEP*/*ABA1* and 2 *NCED3*) and 8 ABA signal transduction genes (1 *PYL*/*PYR*, *4 PP2C*, 1 *SnRK2.6*/*OST1*, and 2 *AREB*/*ABF*) were identified. In addition, 91 ABA-responsive genes were also identified based on *Arabidopsis* homologous annotation. The 91 ABA-responsive genes contained 23 TFs. Interestingly, the *MYB-7*, *NAC-2*, *NAC-12*, *NF-YA-1*, and *WRKY-1*, which were predicted as the target genes of the ABF TFs (key TFs of the ABA signal pathway) by us, were found in these 23 ABA-responsive TFs. This further confirmed that the method, integrating the expression correlation and the potential binding sites of the promoter analysis to identify the potential regulator of genes, was reliable. It is well known that ABA activates a large number of downstream stress-responsive gene expression to enhance the drought resistance of plants (Fujita et al., 2011; You et al., 2019; Waititu et al., 2021). Among the 91 ABA-responsive genes, we found that most of the non-TF ABA-responsive genes were the stress-responsive genes, primarily involved in drought, salt, cold, heat, and oxidative stress responses (**Supplementary Table S6**). These indicated that the ABA-dependent pathway played a vital role in the higher drought resistance ability of XZSN than that of LK3. Notably, among these ABA biosynthesis, signal transduction, and responsive genes, 2 ABA biosynthesis genes (1 *ZEP*/*ABA1* and 1 *NCED3*), 5 ABA signal transduction genes (3 *PP2C*, 1 *SnRK2.6*/*OST1* and 1 *AREB*/*ABF*), and 63 ABA-responsive genes were also not induced in LK3 at drought treatment for 5 days, but most of them were obviously upregulated in LK3 at drought treatment for 7 days. These suggested that the rapid and strong activation of the ABA-dependent pathway under drought stress led to XZSN having a higher drought resistance ability than LK3. Interestingly, by analyzing the potential transcriptional regulator of ABA biosynthesis genes, we found that many identified ABA-responsive TFs in our study were predicted to be the regulators of ABA biosynthesis genes. This indicated that there might be a feedback regulation between ABA biosynthesis and ABA-responsive TFs.

#### Non-ABA signaling

As signal molecules, CO, H_2_S, NO, and melatonin play important roles in the resistance of abiotic stresses including drought stress (Wang and Liao, 2016; Fancy et al., 2017; Sun et al., 2021; Zhang et al., 2021b; Supriya et al., 2022). In our study, we identified 1 CO biosynthesis gene (*HO1*), 1 H_2_S biosynthesis gene (*OAS-TL*), and 1 melatonin biosynthesis gene (*COMT1*) among the 851 core drought-resistant genes in XZSN and found that *HO1* and *OAS-TL* were specifically induced in XZSN at drought treatment for 5 days. These suggested that non-ABA signaling such as CO, H_2_S, and melatonin participated in drought resistance in Tartary buckwheat, and the CO and H_2_S signaling involved in drought resistance in Tartary buckwheat might be genotype-dependent.

#### Water holding

A large number of studies have demonstrated that the stomatal closure and the cuticular wax biosynthesis of the leaves play the most crucial role in preventing leaf water loss under drought stress (Shepherd and Wynne Griffiths, 2006; Lee and Suh, 2015; Xue et al., 2017; Nadeem et al., 2019). Consistent with the physiological analysis result that the drought-tolerant genotype XZSN had significantly higher RWC than the drought-susceptible LK3, we identified 7 ABA-dependent and 1 ABA-independent stomatal closure genes in the 851 core drought-resistant genes in XZSN. The ABA-dependent stomatal closure genes included 2 ABA transporter genes which are homologs of *AtABCG22* and *OsABCG5* with the function of transporting ABA into the leaf guard cell to regulate stomatal closure (Kuromori et al., 2011; Matsuda et al., 2016), 1 NADPH/respiratory burst oxidase gene (*RBOHF*) which mediated ABA-dependent H_2_O_2_ production for stomatal closure (Postiglione and Muday, 2020), 1 anion transporter gene (*ALMT12*) (Meyer et al., 2010), 2 nucleocytoplasmic lectin genes (*EULS3*) (Van Hove et al., 2015), and 1 receptor-like cytoplasmic kinase gene (*CDL1*) (Kim et al., 2018). The ABA-independent stomatal closure genes consisted of 1 hexokinase gene (*HXK1*) (Lugassi et al., 2019). In addition, a total of 10 (*LACS1*, *KCS1*, *KCS6*, *HCD*, 2 *CER1*, 2 *WAX2*, *CER4*, and *WSD1*) and 2 (2 *LTPG2*) homologous genes of cuticular wax biosynthesis and transport were also identified in the 851 core drought-resistant genes (Lee and Suh, 2015). Notably, in LK3, most of the stomatal closure and cuticular wax biosynthesis genes were not induced by the early drought stress (drought treatment for 5 days), but they were significantly upregulated by the later drought stress (drought treatment for 7 days). All these indicated that both stomatal closure and cuticular wax biosynthesis participated in the prevention of Tartary buckwheat leaf water loss under drought stress, and the quick and strong induction of the stomatal closure and the cuticular wax biosynthesis in XZSN contributed to its higher leaf water holding and drought resistance ability than LK3. To date, little is known about the regulator of stomatal closure genes. In our study, we identified that MYB-3, MYB-6, MYB-related-1, HD-ZIP-1, and HD-ZIP-2 TFs might be the major regulators of stomatal closure of Tartary buckwheat under drought stress because they were the potential upstream regulators of most stomatal closure genes. In *Arabidopsis*, the MYB TFs were identified to be the major regulators of wax biosynthesis and transport (Lee and Suh, 2015). In our study, we found that 4 MYB TFs (MYB-1, MYB-5, MYB-6, and MYB-7) might be the major regulators of wax biosynthesis and transport of Tartary buckwheat under drought stress on account that they are the putative upstream regulators of 8, 8, 7, and 7 wax biosynthesis and transport genes. This suggested that MYB TFs have conserved roles in wax biosynthesis and transport in different plants. Notably, MYB-6, which is an ABA-responsive TF, was predicted to be the regulator of 7 stomatal closure and 7 wax biosynthesis and transport genes, implying that it might be the major regulator of the water retention genes under drought stress.

#### Reactive oxygen species scavenging

Enzymatic and non-enzymatic antioxidants are the major defensive mechanism of ROS in plants under drought stress (Nadeem et al., 2019). The enzymatic antioxidants include CAT, superoxide dismutase (SOD), ascorbate peroxide (APX), glutathione peroxidase (GPX), glutathione reductase (GR), dehydroascorbate reductase (DHAR), monodehydroascorbate reductase (MDHAR), and glutathione s-transferase (GSTFs), and the non-enzymatic antioxidants comprise glutathione, ascorbic acid, flavonoids, and so on (Nadeem et al., 2019). In our study, we identified 6 genes encoding the enzymatic antioxidants (1 APX, 2 PRXs, and 3 GSTFs) and 4 non-enzymatic antioxidants consisting of 2 ascorbic acid (*PGM2* and *MIOX1*) and 2 flavonoid (*CHI* and *F3H*) biosynthesis genes among the 851 core drought-resistant genes in XZSN. Among these identified ROS scavenging genes, most of them were not induced in LK3 at drought treatment for 5 days but were upregulated in LK3 at drought treatment for 7 days. This was consistent with the physiological analysis results that the drought-tolerant genotype XZSN had significantly lower MAD content and REL than the drought-susceptible LK3 and suggested that the quick, strong, and specific activation of the enzymatic and non-enzymatic ROS defensive mechanisms played an important role in the high drought resistance ability of XZSN.

#### Osmotic adjustment

Under drought stress, plants quickly synthesize a large number of osmotic adjustment solutes such as amino acid, polyamine, and soluble sugar to protect cells from osmotic damage (Zhu, 2016; Todaka et al., 2017). In our study, we identified many key genes that participated in the biosynthesis of amino acid, polyamine, and soluble sugar among the 851 core drought-resistant genes in XZSN (**Supplementary Table S10**). In amino acid biosynthesis, 2 proline-related genes were identified, which was consistent with the physiological results that XZSN had higher PC than LK3 under stress drought. Furthermore, 10 genes, involved in glutamine, glutamate, aspartate, glycine, cysteine, and methionine biosynthesis, as well as in valine, leucine, and isoleucine biosynthesis, also were identified. In rice, metabolome analysis reveals that the contents of many amino acids are significantly increased under drought stress (Todaka et al., 2017). All these suggested that most amino acids except proline might also play crucial roles in drought stress resistance. In *Arabidopsis*, the *ADC1* encodes the rate-limiting enzyme of PA biosynthesis and plays a crucial role in abiotic stress resistance (Soyka and Heyer, 1999; Capell et al., 2004). In our study, the homologs of *ADC1* were found among the 851 core drought-resistant genes in XZSN, which suggested that the PA might play an important role in drought stress resistance. For soluble sugar biosynthesis, we identified 2, 5, and 2 genes involved in sucrose biosynthesis, raffinose biosynthesis, and starch degradation among the 851 core drought-resistant genes in XZSN. Notably, these raffinose biosynthesis genes were involved in almost all steps of the raffinose biosynthesis pathway. This indicated that raffinose might be the most important soluble sugar involved in drought stress resistance of Tartary buckwheat. Among these identified osmotic adjustments, most were not induced in LK3 at drought treatment for 5 days but were upregulated in LK3 at drought treatment for 7 days. These indicated that the quick accumulation of compatible solutes contributed to the higher drought resistance ability of XZSN than LK3. A transcriptional regulatory network analysis of these osmotic adjustment genes identified many TFs to be their potential upstream regulators. Among these TFs, the MYB-3, MYB-6, HD-ZIP-1, HD-ZIP-2, HD-ZIP-3, MYB-2, MYB_related-1, and NAC-1 were putative as the regulators of 17, 16, 16, 16, 14, 13, and 12 out of the 21 osmotic adjustment genes. These indicated that the 8 TFs might be the major regulators of the accumulation of osmotic adjustment solutes in Tartary buckwheat under drought stress.

## Conclusion

In the present study, we carried out comparative physiological and transcriptomic analyses between the drought-tolerant and drought-sensitive genotypes at the reproductive stage under the field environment of drought stress to gain insight into the molecular mechanism of genotype-dependent drought resistance of Tartary buckwheat. We identified the key genes and regulatory pathways associated with drought tolerance in Tartary buckwheat. Our results showed that the quick, strong, and special transcriptional reprogramming of a large number of genes related to stress resistance in the drought-tolerant genotype in the early stage of drought stress contributed to its higher drought resistance, through decreasing leaf water loss, activating osmotic protection, ROS scavenging, enhancing cell damage protection mechanisms, and so on. These results enhanced our understanding of the physiological and molecular mechanisms of the genotype-dependent drought resistance of Tartary buckwheat and highlighted the possible candidate genes that might play important roles in the drought tolerance of Tartary buckwheat.

## Data availability statement

The original contributions presented in the study are publicly available. This data can be found here: NCBI, PRJNA859365

## Author contributions

H-YL designed the study, performed the data analysis, revised the original draft, and supervised the study. H-LM performed the experiments and wrote the original draft. P-YS and J-RW performed the experiments and data analysis. X-QS, C-ZZ, and TF participated in the experiments. Q-FC revised the original draft. All authors contributed to the article and approved the submitted version.

## Funding

This work was supported by the Joint Fund of the National Natural Science Foundation of China and the Karst Science Research Center of Guizhou Province (U1812401), the Opening Foundation of the Key Laboratory of Plant Resources Conservation and Germplasm Innovation in Mountainous Region of the Ministry of Education (QianJiaoHeKYZi[2019]035), the Science and Technology Foundation of Guizhou Province (QianKeHeJiChu-ZK[2021]ZhongDian035), the Science and Technology Foundation of Guizhou Province (QianKeHeJiChu[2019]1235), and the Earmarked Fund for China Agriculture Research System (CARS-07-A5).

## Conflict of interest

The authors declare that the research was conducted in the absence of any commercial or financial relationships that could be construed as a potential conflict of interest.

## Publisher’s note

All claims expressed in this article are solely those of the authors and do not necessarily represent those of their affiliated organizations, or those of the publisher, the editors and the reviewers. Any product that may be evaluated in this article, or claim that may be made by its manufacturer, is not guaranteed or endorsed by the publisher.
