# Supplementary figures and images for: Comparative physiological, transcriptomic, and WGCNA analyses reveal the key genes and regulatory pathways associated with drought tolerance in Tartary buckwheat
Source: Front Plant Sci. 2022 Oct 3;13:985088. doi: 10.3389/fpls.2022.985088 (PMC9575659; doi:10.3389/fpls.2022.985088)

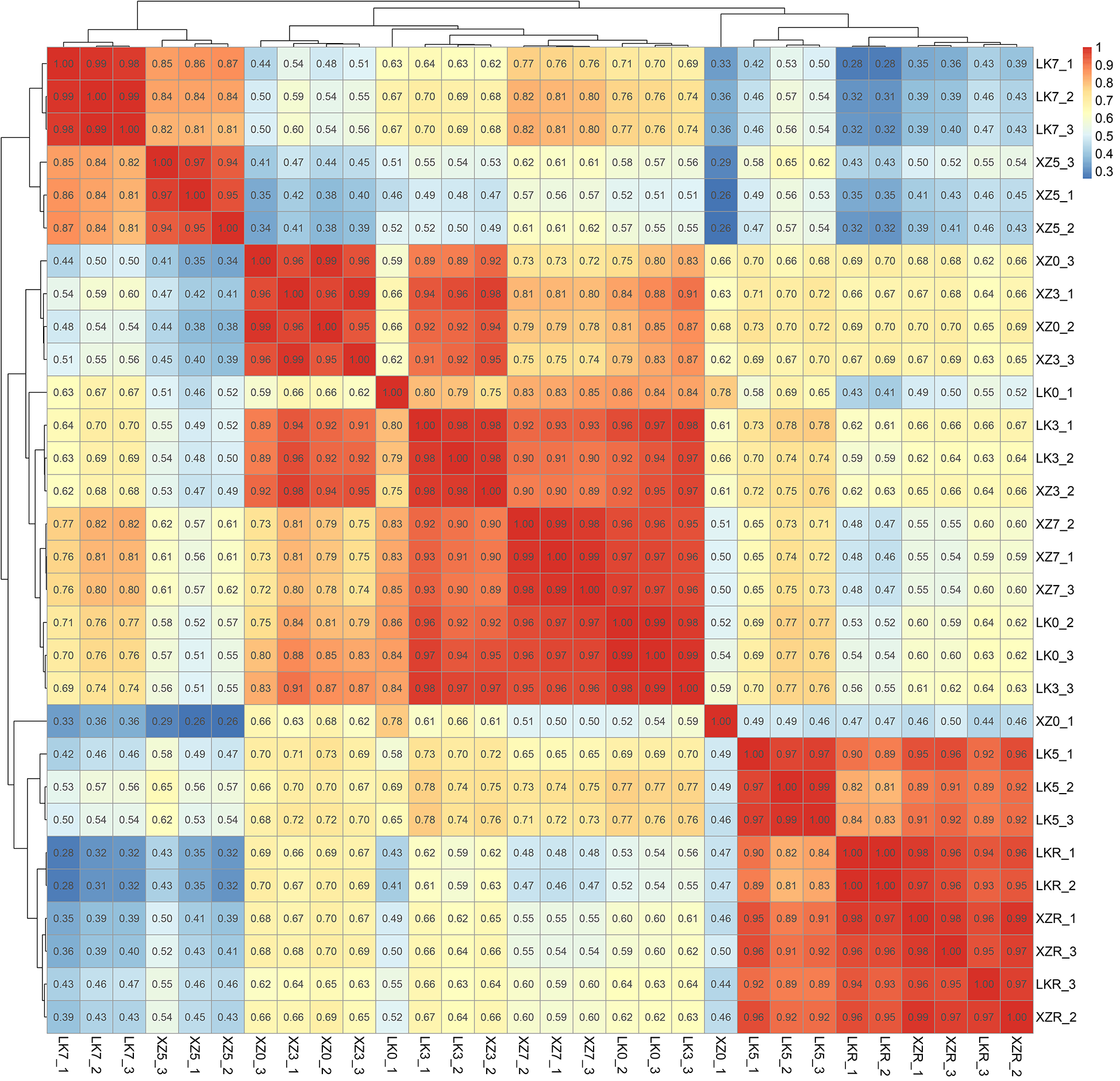

Supplement: Supplementary file 1 [file Image_1.tif]

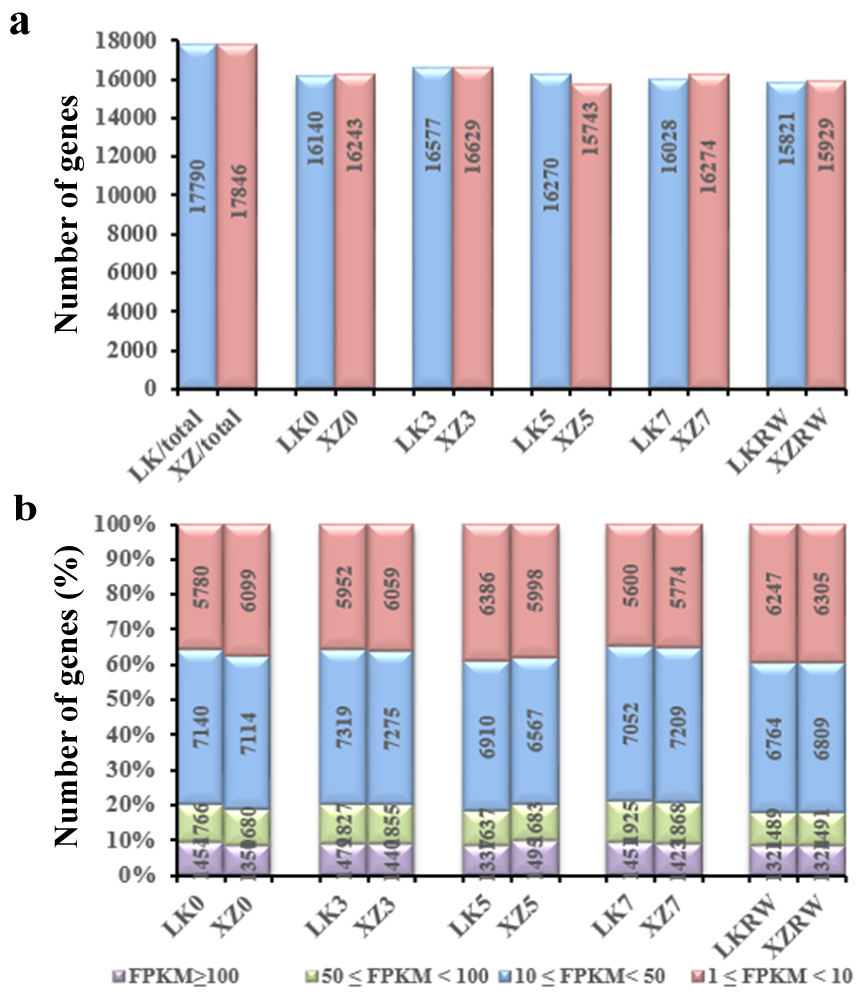

Supplement: Supplementary file 2 [file Image_2.tif]
