# Supplementary material for: Comparative physiological, transcriptomic, and WGCNA analyses reveal the key genes and regulatory pathways associated with drought tolerance in Tartary buckwheat
Source: Front Plant Sci. 2022 Oct 3;13:985088. doi: 10.3389/fpls.2022.985088 (PMC9575659; doi:10.3389/fpls.2022.985088)
Supplement: Supplementary file 3 [file Table_1.docx]

**Table S1. Primers of sequences for qRT-PCR analysis**

| **Gene ID** | **Forward primer (5`- 3`)** | **Reverse primer (5`- 3`)** |
| --- | --- | --- |
| FtPinG0007062500.01 | GTATCCACGACATAACCACC | GTGTACAGGGATCCTTGGTT |
| FtPinG0005799200.01 | GACATCCATCTCATCCAACC | CCACCATACCTTCAAGCAAC |
| FtPinG0009700500.01 | CTGATTGGAAGTCCATCTCG | CATTGGATGGTGACAACGAG |
| FtPinG0000334100.01 | GAGTGCAATCGGAGAACATC | GAGGTGAATTTCACTGGCGA |
| FtPinG0000281400.01 | TCTTGATCCTTCCTCGTACC | TGGTGTACTTCGATCAGGTC |
| FtPinG0004759200.01 | GAAGAACTCTGAGCTGGATG | CCGTAACTAGATCCAGATCC |
| FtPinG0003023300.01 | GCAGCTTCTGAAGAGGTGTT | GGACTTCCCAGATTAGATCC |
| FtPinG0001748800.01 | AAGAACATGGCTGAACCCAC | TGCAGCAAACCTCTATGCAC |
| FtPinG0001027400.01 | ACGTCAAGTGGAAGTGTGGT | AAGGAGCAAAGCATCCAGAG |
| FtPinG0003045600.01 | GGTATGGTAAGGAGCTAGTG | CAAGCAACCTCACAAGCATG |
| FtPinG0000667700.01/HLK | ATGTTGTGGGACGTGTGGTT | AAGCGATCCTCTGGTGATAG |
